# Optimization of hole quality in drilling of direct hot-pressed Al/SiC composites using Taguchi method

**DOI:** 10.1038/s41598-026-42714-6

**Published:** 2026-03-15

**Authors:** Gokhan Basar, Oguzhan Der, Funda Kahraman, Sinan Önder

**Affiliations:** 1 Department of Industrial Engineering, Faculty of Engineering and Natural Sciences, Osmaniye Korkut Ata University, Osmaniye, 80010 Türkiye; 2 Marine Engineering Department, Bandirma Onyedi Eylul University, Balikesir, 10200 Türkiye; 3 Department of Mechanical Engineering, Faculty of Engineering, Tarsus University, Mersin, 33400 Türkiye; 4 Department of Machinery and Metal Technologies, Torbalı Vocational School, Dokuz Eylul University, İzmir, 35860 Türkiye

**Keywords:** Aluminium matrix composites, Direct hot-pressing, Drilling performance, Taguchi method, Variance analysis, Regression analysis, Engineering, Materials science

## Abstract

In this study, aluminium (Al) matrix composites reinforced with 0, 5, and 10 vol% silicon carbide (SiC) particles were fabricated by direct hot-pressing under 35 MPa pressure at 600 °C for a holding time of 5 min. The morphological characteristics of the initial Al and SiC powders were examined using scanning electron microscopy. The fabricated composites were characterized through density measurements, microstructural analysis, X-ray diffraction, and microhardness testing. Microstructural observations confirmed a homogeneous distribution of SiC particles within the Al matrix. Although the relative density decreased with increasing SiC content, microhardness increased due to restricted dislocation motion induced by the hard ceramic reinforcement. The cutting speed, feed rate, point angle, and reinforcement ratio were selected as control factors in the drilling process of Al/SiC composites. In contrast, thrust force, surface roughness, deviation from diameter, and deviation from circularity were selected as performance indicators. The Taguchi method was used to determine the optimal experimental conditions for hole performance indicators. The effects of drilling parameters on the drill bit, hole quality, and chip morphology were investigated. The contribution ratios of the control factors on the responses were determined using analysis of variance. The feed rate was found to be the most effective control factor on hole quality. Regression analysis was applied to establish a mathematical relationship between the control factors and the responses. The R^2^ values obtained from the regression equations were found to be quite high. Finally, confirmation experiments conducted with the determined optimal parameter sets have proven the validity of the models by yielding statistically significant results within a 95% confidence interval.

**Supplementary Information:**

The online version contains supplementary material available at 10.1038/s41598-026-42714-6.

## Introduction

Aluminium matrix composites (AMCs) offer a blend of the relatively low density, great specific strength, good corrosion resistance, and high thermal conductivity signatures generally found in aluminium (Al) alloys with the utmost hardness, stiffness, and wear-resistance marks possessed by their ceramic reinforcements^1^. Therefore, AMCs have won large spotlight for the advanced engineering applications with the aerospace^2^, notably automotive^3^, defence forces^4^, and electronics^5^ requiring light structures with profoundly enhanced mechanical and tribological properties. Silicon Carbide (SiC) particles are widely used as a filling material because they greatly enhance the hardness, elastic modulus, and wear resistance of robotically deposited Al matrices, whilst maintaining a good thermal stability^6^.

AMCs can be produced using both liquid-state routes (e.g., stir casting and infiltration) and solid-state routes based on powder metallurgy^7^. Compared with liquid processing methods, powder metallurgy offers important advantages, including better control over reinforcement distribution, reduced segregation, lower processing temperatures, and a minimized risk of undesirable interfacial reactions^8,9^. Within powder metallurgy techniques, direct hot-pressing (DHP) is particularly attractive, as it enables simultaneous application of pressure and temperature, leading to rapid densification, improved interfacial bonding, limited grain growth, and shorter processing times^10^. These characteristics make DHP an effective and reliable method for producing high-quality Al/SiC composites with controlled microstructural features. It should be noted that the manufacturing route plays a critical role in determining the machinability of Al/SiC composites. Compared with conventionally cast or stir-processed composites, DHP generally promotes a more homogeneous reinforcement distribution, improved matrix–particle interfacial bonding, and reduced segregation, which are expected to contribute to more stable cutting behaviour and improved hole quality during drilling^11,12^. A direct, head-to-head comparison of these processing routes under identical drilling conditions would therefore be a valuable subject for future investigations.

Compared with stir-cast Al/SiC composites, DHP materials generally exhibit more stable machinability due to their homogeneous reinforcement distribution, reduced porosity, and improved matrix–particle interfacial bonding, which contribute to lower cutting instability and improved hole quality^13^. While spark plasma sintering can produce even higher densification and finer microstructures, the resulting increase in hardness may intensify tool abrasion, whereas DHP offers a balanced combination of enhanced mechanical properties and stable drilling performance^14^. The DHP route promotes a homogeneous distribution of SiC particles, strong matrix–reinforcement interfacial bonding, and reduced porosity, all of which play a decisive role in governing the cutting mechanics during drilling. These microstructural characteristics contribute to more stable material removal, lower thrust force fluctuations, and improved surface integrity and geometric accuracy of drilled holes compared with composites produced by less controlled fabrication routes.

Al/SiC composites have been found beneficial in industrial applications. Structures of these are later said to machine for final assembly or fastening requirements such as in drilling work^15^. Drilling of Al/SiC composites is found to be more difficult than monolithic Al alloys due to the presence of SiC particles which are hard and abrasive in nature^16,17^. Consequently, the drilling action involves SiC particles that fairly promote tool wear, increase drill & drill performance load, form built-up edge (BUE), reduce hole surface integrity, and compromise on dimensional accuracy/performance^18,19^. This necessitates studies to understand and optimize the drilling action of Al/SiC composites for the specification of hole quality and productive reliability in the industrial applications.

Hole quality is a multidimensional concept that directly affects assembly precision, load-bearing capability of fasteners, and fatigue performance of drilled components^13^. In drilling operations, hole quality is strongly influenced by cutting parameters such as cutting speed, feed rate, tool geometry (especially point angle), and material-related factors such as reinforcement content^20^. Variations in these parameters alter the cutting mechanics, heat generation, and chip formation behaviour, which in turn affect critical hole quality characteristics^21^. In the present study, hole quality is evaluated using thrust force (Fz), surface roughness (Ra), diameter deviation (DD), and circularity deviation (CD), providing a comprehensive assessment of both surface integrity and geometric accuracy.

A large body of literature has examined the machinability and drilling behaviour of AMCs; however, these studies predominantly concentrate on a limited set of output responses, most Fz, torque, or Ra, without extending the analysis to geometric accuracy of the drilled holes. For instance, Chakraborty et al. demonstrated that drilling performance of Al-MMCs is strongly governed by feed rate, cutting speed, and point angle, yet their optimization framework mainly targeted Fz, Ra, and burr height, neglecting dimensional and form deviations such as diameter and circularity errors^22^. Similarly, studies on the machinability of powder-metallurgy-based Al hybrid composites have largely focused on tool wear and Ra during milling or drilling, emphasizing the abrasive effect of hard ceramic reinforcements while providing limited insight into hole geometry integrity^23^. Although reinforcement content is known to significantly influence hardness, wear resistance, and microstructural homogeneity in Al-based composites produced by advanced sintering routes, including microwave sintering and hot-pressing, most investigations remain confined to mechanical or tribological performance rather than post-sintering machinability outcomes^24,25^. Even in recent studies on hybrid and particle-reinforced Al composites, comprehensive drilling assessments that simultaneously address Fz, Ra, DD, and CD are rarely reported, despite their critical importance for assembly precision and functional performance^26^. Consequently, there exists a clear research gap for an integrated evaluation of both mechanical and geometrical hole quality characteristics, particularly for Al/SiC composites fabricated via DHP, which motivates the need for a more holistic and multi-response optimization approach in drilling studies. This study distinguishes itself by jointly examining the effects of the manufacturing route, drilling parameters, and multi-response hole quality in Al/SiC composites. Unlike most previous studies that focus on a limited number of machinability outputs, Fz^27^, Ra^28^, DD^13^, and CD^13^ are simultaneously evaluated for composites produced by DHP. This integrated approach enables a quantitative linkage between DHP-induced microstructural integrity and drilling-induced surface and geometric accuracy.

To address this gap, the present study systematically investigates four fundamental hole quality responses (Fz, Ra, DD, and CD) within a single experimental framework. In the first stage, Al/SiC composites containing different SiC volume fractions were produced using DHP. Subsequently, density measurements, microstructure analysis using scanning electron microscopy (SEM) and energy-dispersive X-ray spectroscopy (EDX), X-ray diffraction (XRD) analysis, and microhardness testing were performed on the composites. In the second stage, optimal experimental conditions were determined using the Taguchi method to minimize the hole performance indicators. The three-dimensional surface graphs were used to examine how different drilling parameters impacted the indicators of hole quality. The study examined how different drilling parameters affected three aspects which included the drill bit performance and the quality of the drilled hole and the characteristics of chip production. The third stage of the project used variance analysis to determine how much control factors affected the different measured responses. The study used regression analysis to create a mathematical model which ties together control factors with their corresponding results. The research team performed their final validation tests by using the best experimental conditions which had been established for testing responses, and they calculated confidence intervals. This research study which evaluates four machinability indicators simultaneously with Fz, Ra, DD, and CD assessment presents a unique approach to analyzing the DHP Al/SiC composite drilling process. The combination of multi-response Taguchi optimization with variance analysis and regression modeling together with confidence interval validation creates a new method for measuring how processing route and reinforcement ratio interact with drilling parameters to affect both surface integrity and geometric accuracy of drilled holes.

## Materials and methods

### Manufacturing of Al/SiC composites

Pure Al (> 99%) and silicon carbide (SiC) powders (99.95%) with average particle sizes (D50) of 43.3 µm and 5.08 µm, respectively, were used for specimen preparation. The morphological characteristics of the starting powders were examined using a FEI Quanta 650 Field Emission SEM. The manufacturing route for the pure Al and the composites reinforced with 5 and 10 vol% SiC consisted of weighing the powders using a precision balance, homogenizing the powder mixtures in a Turbula mixer, and consolidating them by DHP. The samples were fabricated in a PLC-controlled DHP unit equipped with a direct resistance heating system under an argon atmosphere at a pressure of 35 MPa and a temperature of 600 °C, with a holding time of 5 min. In the production of Al/SiC composites via the DHP process, a sintering temperature of 600 °C facilitates matrix softening and enhances powder compressibility, thereby contributing to an increase in relative density^29^. A pressure of 35 MPa and a sintering time of 5 min were optimized to overcome the constraining effect of ceramic reinforcements on matrix deformation and to establish good interfacial bonding between the matrix and the reinforcement, thus minimizing structural defects^13^.

### Density, microstructure, and hardness

The experimental densities ($\rho_{e}$) of the specimens were measured in accordance with ASTM B962-17 standard^30^. The theoretical ($\rho_{t}$) and relative ($\rho_{b}$) densities were computed using Eqs. (1) and (2), respectively^13^.

1 $$\rho_{t} = \rho_{m} \times V_{m} + \rho_{r} \times V_{r}$$

Here, $\rho_{m}$ and $\rho_{r}$ denote the density of the matrix and particle powder, respectively. In contrast, $V_{m}$ and $V_{r}$ denote the volume ratio of the matrix and particle, respectively.

2 $$\rho_{b} = \frac{{\rho_{e} }}{{\rho_{t} }} \times 100$$

For microstructural and EDX analyses, the samples were sequentially ground using SiC abrasive papers with grit sizes of 240, 400, 600, 800, and 1200. Subsequently, polishing was performed using diamond suspensions with particle sizes of 6, 3, and 1 μm on a polishing cloth. The polished samples were then etched for 50 s using a modified Keller’s reagent consisting of 10 mL HNO_3_, 1.5 mL HCl, 1 mL HF, and 87.5 mL distilled water. After etching, the samples were rinsed and dried. Microstructural observations and EDX analyses were carried out using SEM (FEI QUANTA FEG 250). XRD analysis was performed using a PANalytical EMPYREAN diffractometer. The XRD patterns were recorded in the 2θ range of 10°–90° at a scanning rate of 2° min⁻^1^ using Cu Kα radiation (λ = 1.5406 Å) operated at 40 mA and 45 kV. Phase identification was conducted using the HighScore Plus software (Malvern Panalytical HighScore)^31^ in conjunction with the Inorganic Crystal Structure Database (ICSD)^32^. The Vickers microhardness of the specimens was measured using a Future-Tech FM-700 tester. The tests were conducted with a diamond pyramid indenter having an apex angle of 136°, under a load of 50 gf applied for a dwell time of 10 s.

### Drilling process

**Fig. 1:**
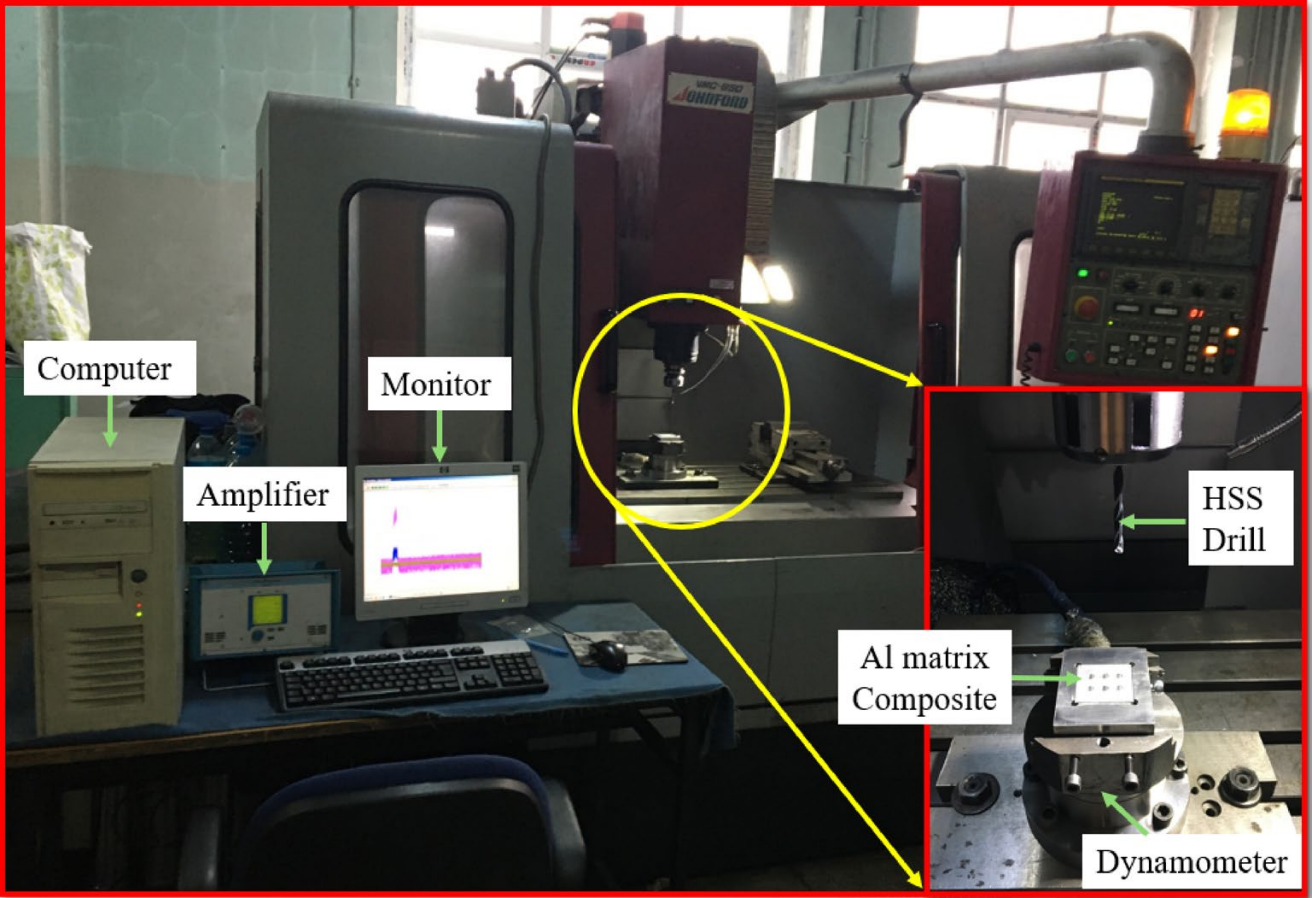
Experimental setup used for the drilling tests.

Drilling tests of the composites were carried out on a Johnford VMC-850 CNC machining center. Drilling experiments were carried out on 10 mm thick, 40 × 40 mm square specimens using 5 mm diameter High-speed steel (HSS) twist drill, and holes with a total length of 10 mm were drilled. In the experiments, HSS twist drill bits with tip angles of 100°, 118°, and 136° were used. HSS drills were intentionally used to establish a baseline drilling condition and to clearly reveal the influence of cutting parameters and SiC reinforcement on hole quality. In addition, HSS tools are still commonly employed in industrial drilling of Al-based composites at moderate cutting speeds, and their lower wear resistance enables a more sensitive assessment of the abrasive effects of SiC particles. The non-standard point angles (100° and 136°) were obtained by regrinding DIN 338 standard drills supplied by Makine Takım Endüstrisi A.Ş. within a tolerance of ± 1–2°. To ensure precise positioning of the specimens on the dynamometer, a custom-designed clamping fixture was manufactured. This fixture was integrated with the dynamometer mounted on the machine table, enabling the drilling experiments to be conducted under rigid and stable conditions (Fig. 1).

In the experimental study, cutting speed (Vc, m/min), feed rate (f, mm/rev), point angle (PA, °), and reinforcement ratio (RR, vol.%) were selected as control factors, while Fz (N), Ra (µm), DD (mm), and CD (mm) were evaluated as hole quality characteristics. The levels of drilling parameters were determined based on a comprehensive literature review and the recommendations of the cutting tool manufacturer and are provided in Table 1. To minimize the number of experiments required for drilling Al/SiC composites, a mixed-level Taguchi L_18_ orthogonal array was employed.

**Table 1:** Control factors and levels used in drilling experiments.

Control factors	Levels		
	1	2	3
Cutting speed (m/min)	25	50	–
Feed rate (mm/rev)	0.08	0.16	0.24
Point angle (°)	100	118	136
SiC reinforcement ratio (vol. %)	0	5	10

### Measurement of hole quality characteristics

**Fig. 2:**
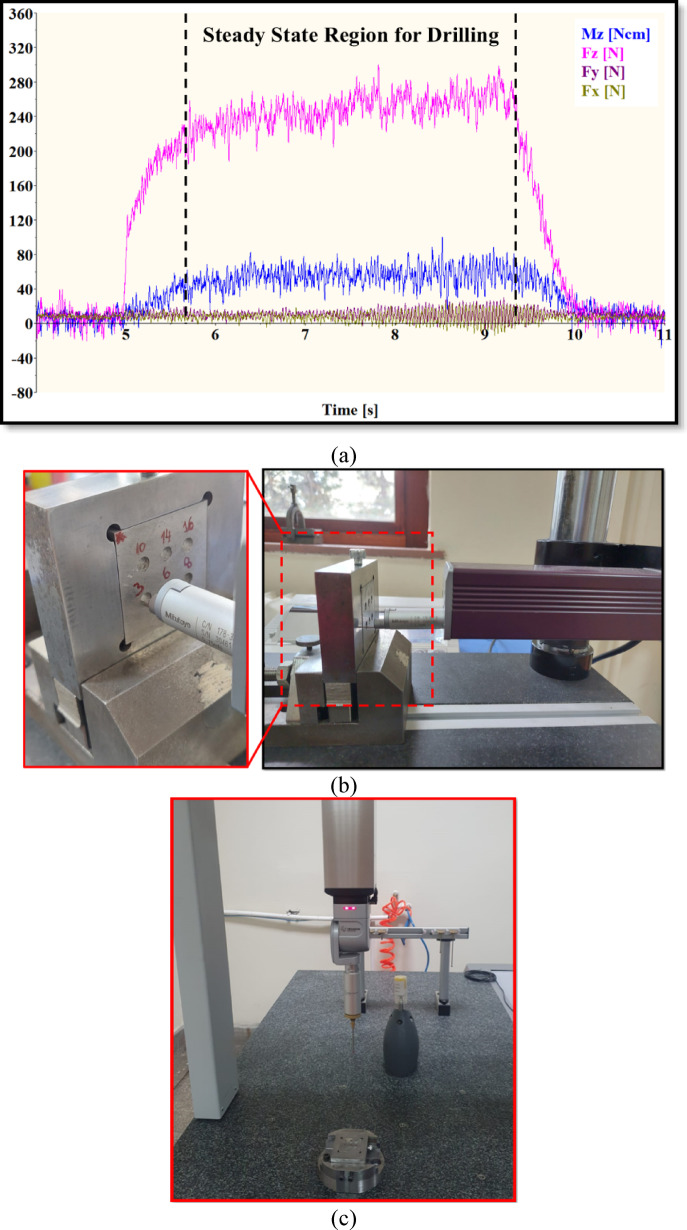
Measurement of the response variables: (**a**) Fz, (**b**) Ra, and (**c**) DD and CD.

Fz measurements were carried out using a data acquisition (DAQ) system consisting of a Kistler 9257A piezoelectric dynamometer integrated into the CNC machining center and a Kistler 5070-A multichannel charge amplifier for signal conditioning. The acquired raw signals were processed using DynoWare software, and the Fz values were calculated by averaging the regions exhibiting steady-state behavior on the force–time plots. To ensure experimental repeatability, each drilling condition was repeated three times. For the evaluation of hole quality, Ra measurements were performed using a Mitutoyo SJ-410 surface profilometer, with four measurements taken for each specimen. DD and CD were determined using a Hexagon Global Performance coordinate measuring machine (CMM). CMM data were taken during an automated probe movement generated measurement, at the three different positive depths along the Z-axis (− 2, − 5, and − 7 mm measurements) and with regard to the measure feature for surface height on the specimen plane. A matching example image is shown in Fig. 2 for the hole quality characteristics measurement procedure. The Dino-Lite AM4113T digital optical microscope and Dino Capture 2.0 software were used to examine drill bits, hole images, and chip morphology.

### Taguchi method

In this study, the Taguchi method was used because it allows the optimization of drilling parameters with a smaller number of experiments. The use of orthogonal arrays significantly reduces experimental time and cost compared to traditional full factorial designs. In addition, signal-to-noise (S/N) ratio analysis provides a reliable optimization result by focusing on performance consistency and mitigating the effects of uncontrollable noise factors^33,34^. Moreover, the Taguchi method accentuates the robust nature of the process against the performance variability originating from uncontrollable noise elements^35^. In this model, loss functions allows calculating the deviation between measured process aspects and their planned target values to give a notion about quality loss that comes with variation^36^. The calculated loss function values are subsequently transformed into S/N ratios, which serve as objective functions for optimization and enable the assessment of both mean performance and robustness simultaneously. Depending on the optimization objective, S/N ratios are classified into three categories: smaller-is-better, larger-is-better, and nominal-is-best^37,38^. In the present study, since all investigated hole quality characteristics are required to be minimized, the smaller-is-better criterion was adopted, and the S/N ratios were calculated using Eq. (3).

3 $$\frac{S}{N} = - 10log\left( {\frac{1}{n}\mathop \sum \limits_{i = 1}^{n} y_{i}^{2} } \right)$$

Here, $y_{i}$ denotes the observed value of the response for the $i$ th experiment, and n represents the total number of experimental observations^38^.

## Results and discussion

### Properties of powders

**Fig. 3:**
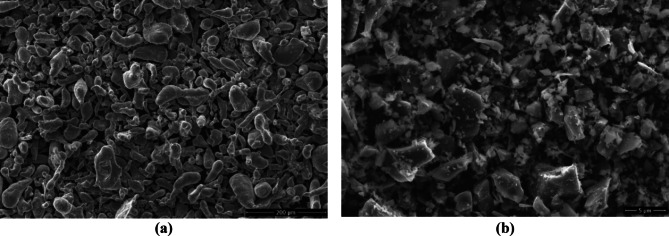
SEM images of the starting powders at different magnifications: (**a**) Al and (**b**) SiC.

The morphological characteristics of the powders are presented in Fig. 3. According to the SEM micrographs, the Al powders exhibit relatively coarse, irregular, and elliptical or flake-like morphologies, whereas the SiC powders consist of much finer particles with angular shapes and sharp edges. The D50 values of 43.3 µm for the Al powders and 5.08 µm for the SiC powders indicate that the reinforcement phase is approximately eight times smaller in size than the matrix phase. This pronounced size difference facilitates the accommodation of SiC particles within the interstitial spaces between Al grains, increases the matrix–reinforcement interfacial area, and enhances the efficiency of load transfer. Therefore, this morphological structure is expected to positively influence the mechanical properties of the produced composites.

### Density, microstructure, and hardness analysis

Theoretical, experimental, and relative density values ​​of the produced materials are given in Table 2. The results show that the addition of SiC particle reinforcement leads to an increase in theoretical density; however, experimental and relative densities tend to decrease. The decrease in relative density is thought to be due to the increased porosity resulting from the increased reinforcement amount, which makes complete densification more difficult during the hot pressing process.

**Table 2:** Density values of the specimens.

Sample	Theoretical density (g/cm^3^)	Experimental density (g/cm^3^)	Relative density (%)
Al	2.700	2.662	98.58
Al/5SiC	2.726	2.657	97.49
Al/10SiC	2.751	2.647	96.23

**Fig. 4:**
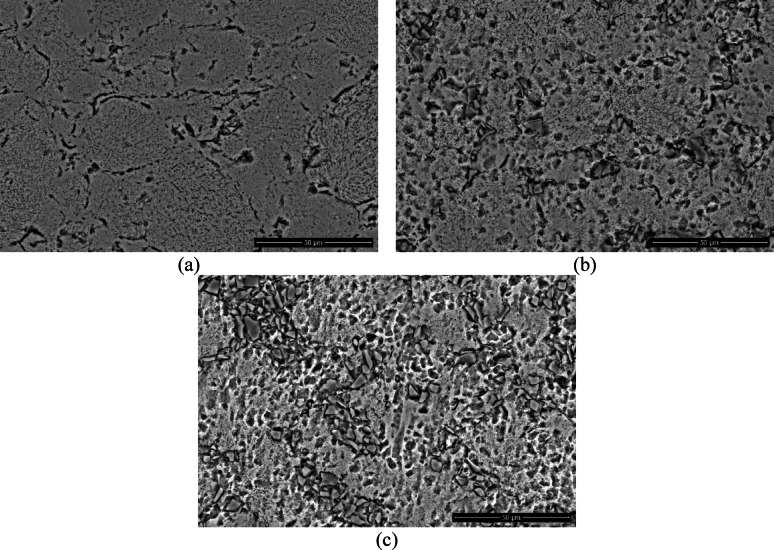
SEM images of the samples at 2500 × magnification (**a**) Al, (**b**) Al/5SiC, (**c**) Al/10SiC.

The SEM micrographs of the samples are presented in Fig. 4. Examination of Fig. 4b and c reveals the presence of SiC ceramic particles embedded within the Al matrix. As the SiC reinforcement content increases, a higher distribution density of the particles within the matrix is observed; however, this is accompanied by an increased tendency toward particle clustering and agglomeration.

**Fig. 5:**
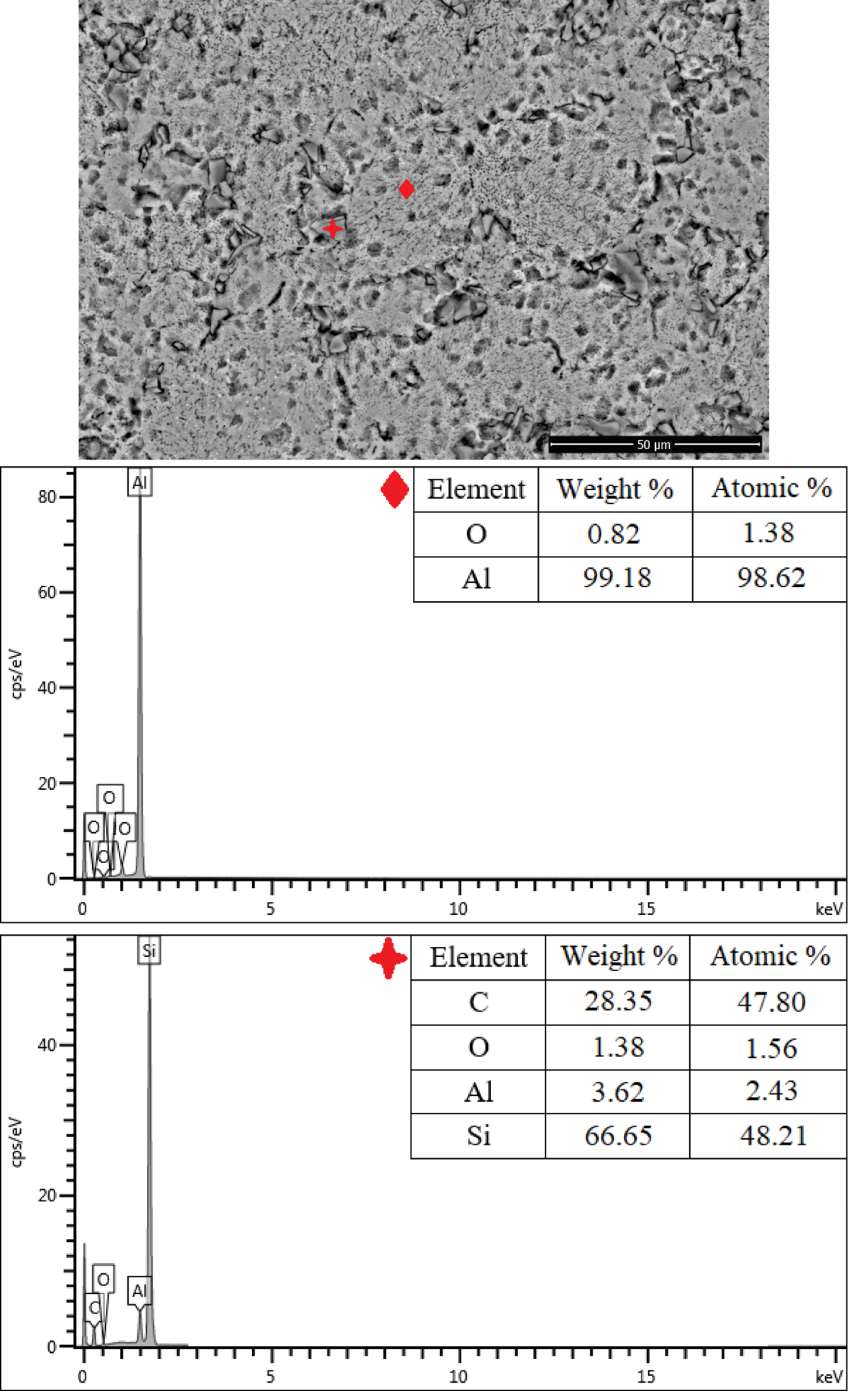
SEM/EDX analysis results of the Al/5SiC composite material.

The EDX/SEM analysis results of the Al/5SiC composite microstructure are presented in Fig. 5. The EDS spectra acquired from the locations marked on the SEM image confirm the expected two-phase structure of the Al-based composite reinforced with 5 vol.% SiC. In the spectrum obtained from the matrix region (diamond-marked point), Al is dominant with an approximate content of 99.2 wt% (98.6 at.%), while only a minor amount of oxygen (~ 0.8 wt%) is detected. This indicates that the analysed region corresponds to an almost pure Al matrix, with the detected oxygen attributed to the naturally formed thin Al_2_O_3_ surface layer, which can be identified by EDS. Similar levels of oxygen signals observed in the matrix of Al/SiC composites have been widely reported in the literature and are commonly associated with the strong affinity of Al for atmospheric oxygen, leading to the formation of a surface oxide layer^39^.

In contrast, the EDS analysis performed on the region corresponding to the SiC particle (plus-marked point) reveals that silicon and carbon are the dominant elements, with weight percentages of 66.65 wt% Si and 28.35 wt% C, and atomic percentages of 48.21 at.% Si and 47.80 at.% C. The nearly stoichiometric Si:C ratio of approximately 1:1 clearly confirms that this phase corresponds to a SiC particle. This observation is consistent with literature findings, where SiC reinforcements in Al/SiC composites are similarly identified by the presence of strong Si and C signals in EDS analyses^39^. As reported by Zapata-Valencia et al.^40^, the detection of minor amounts of Al (3.62 wt%) and oxygen (1.38 wt%) at the same location can be attributed to the interaction volume of the electron beam during EDS analysis, which may partially include the surrounding Al matrix and a possible thin interfacial oxide layer.

In the present study, the absence of any additional elements at the SiC location and the close Si–C atomic ratio indicate that no pronounced interfacial reaction layer developed between the matrix and the reinforcement under the applied powder metallurgy processing conditions. These results suggest that the chemical integrity of the Al matrix was largely preserved and that the SiC reinforcements were successfully embedded within the composite structure as intended. Such findings are in good agreement with previous studies emphasizing that controlled interfacial reactivity is critical for achieving favourable mechanical performance in Al/SiC-based metal matrix composites^39,40^.

**Fig. 6:**
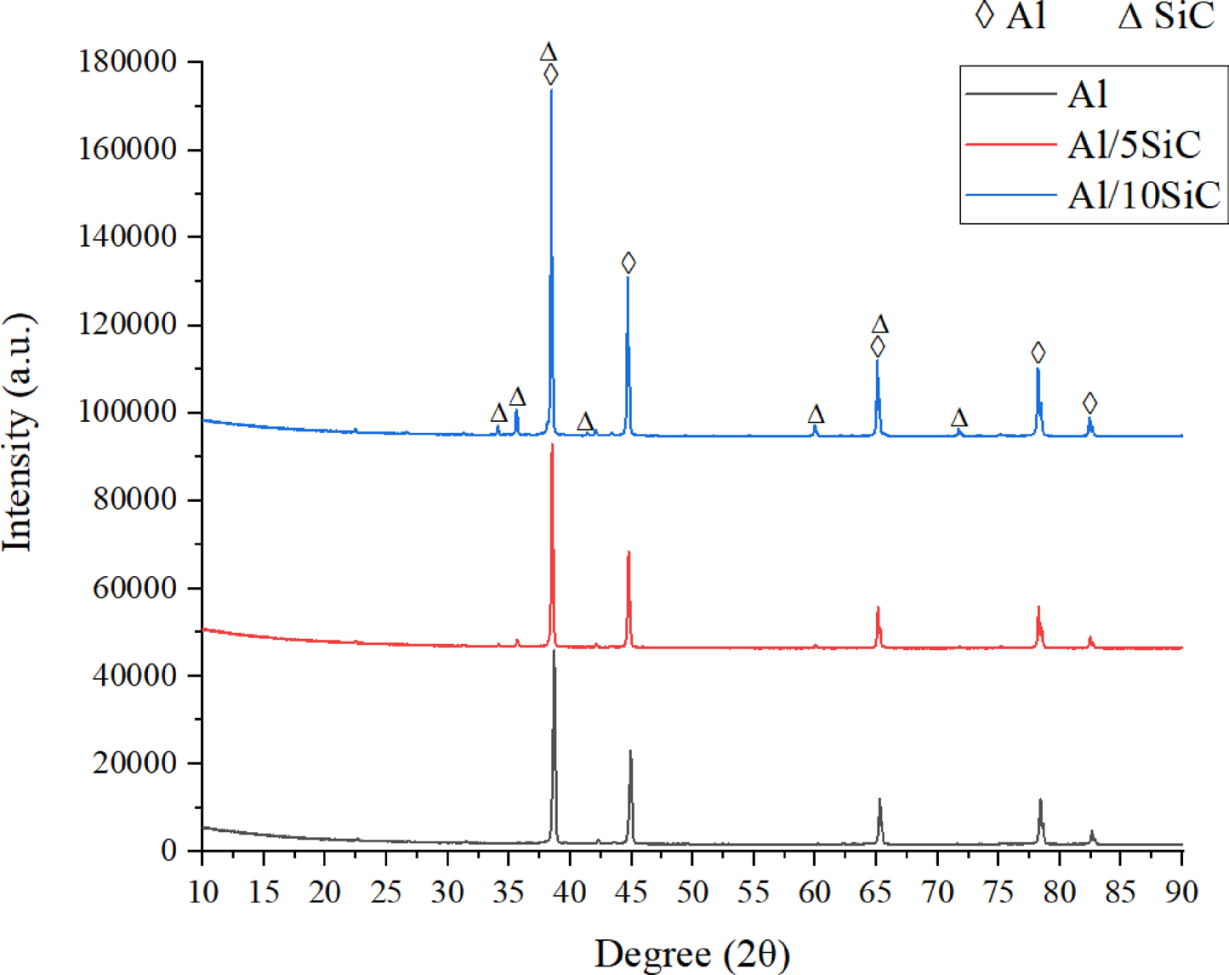
XRD patterns of the samples.

Figure 6 presents the XRD patterns of the sintered pure Al, Al/5SiC, and Al/10SiC composite specimens. In all diffractograms, the dominant and sharp diffraction peaks observed at 2θ ≈ 38.48°, 44.73°, 65.11°, 78.24°, and 82.45° correspond to the (111), (200), (220), (311), and (222) crystallographic planes of the face-centered cubic (FCC) α-Al phase (ICSD: 52255), respectively. The absence of any secondary-phase peaks in the pure Al specimen confirms the high purity of the starting matrix material. With the incorporation of SiC reinforcement, characteristic reflections of the SiC phase (ICSD: 156190), marked by triangular symbols, become apparent, particularly in the Al/10SiC composite. The increase in the intensity and distinguishability of the SiC peaks with increasing reinforcement content up to 10 vol.% indicates that the ceramic phase is successfully distributed within the Al matrix and that a stable composite structure is achieved after sintering. The most intense fundamental diffraction peak of the SiC phase appears at approximately 2θ ≈ 35.65°, while additional secondary reflections are observed at around 34.09°, 41.38°, 60.0°, and 71.75°. SiC reflections reported in the literature at approximately 2θ ≈ 38.13° and 65.6° could not be independently resolved in the present diffractograms due to peak overlap with the high-intensity α-Al matrix peaks located at 38.48° and 65.11°, respectively. Furthermore, the absence of additional diffraction peaks associated with brittle and hydrophilic/hygroscopic carbide phases such as Al_4_C_3_ indicates that no undesirable reaction products formed at the matrix–reinforcement interface under the applied sintering conditions.

**Fig. 7:**
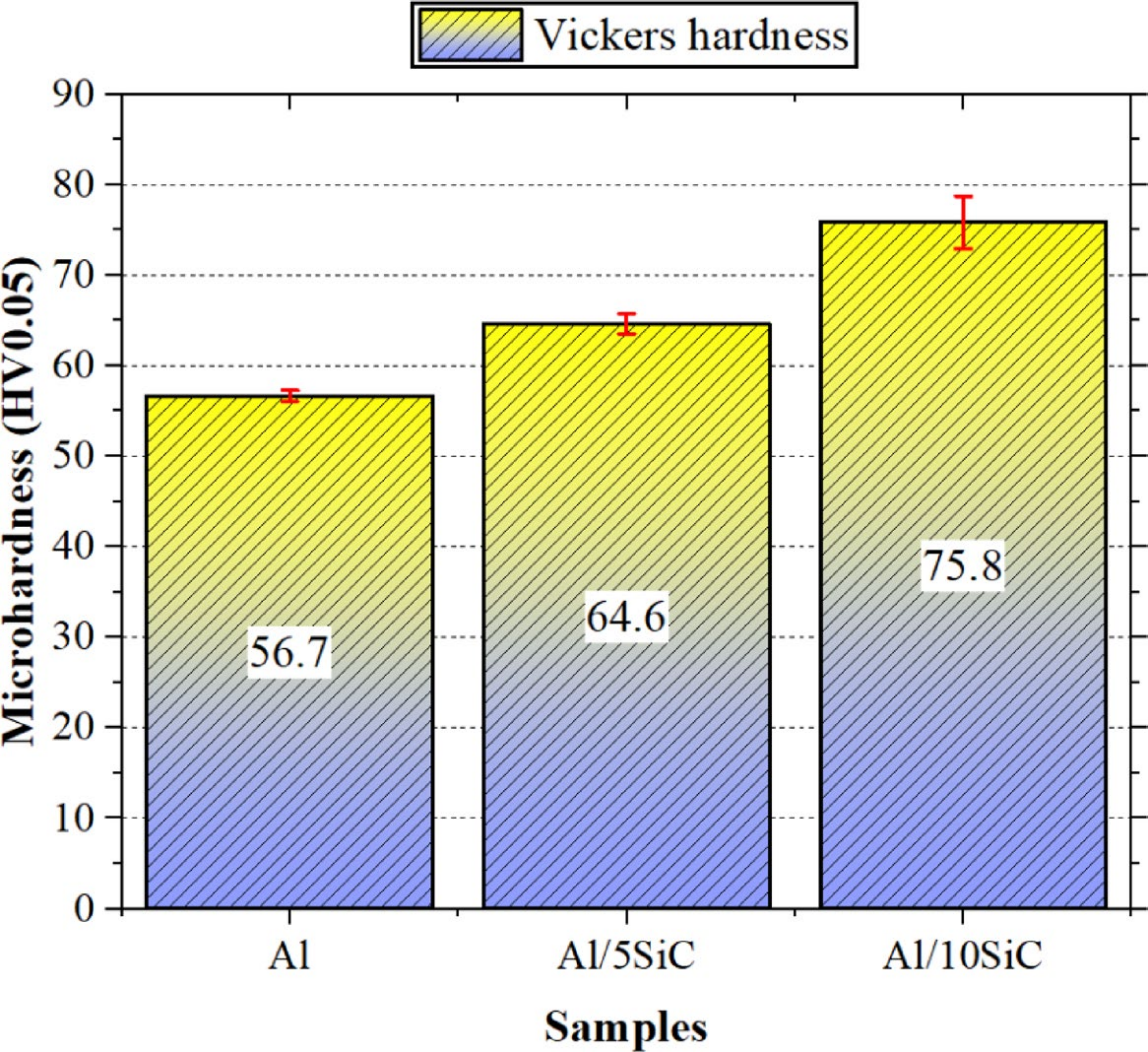
Microhardness results of the specimens.

The microhardness results of the Al/SiC composite materials are shown in Fig. 7. An examination of the figure clearly indicates that SiC particle reinforcement increases the microhardness of the Al/SiC composites. While the average microhardness of the pure Al specimen is 56.7 HV, the values increase to 64.6 HV and 75.8 HV for the composites reinforced with 5 and 10 vol.% SiC, respectively. Accordingly, increasing the SiC content to 5 vol.% results in an approximate hardness increase of 13.9% compared to pure Al, whereas an increase to 10 vol.% SiC leads to a hardness improvement of about 33.7%.

In parallel with this trend, the porosity levels increase from 1.42% in pure Al to 2.51% and 3.77% in the 5 vol.% and 10 vol.% SiC-reinforced samples, respectively. Therefore, despite the pronounced increase in porosity with increasing SiC volume fraction, the effect of SiC addition on microhardness remains dominant and beneficial. This behaviour is micromechanically consistent when the particle sizes and mechanical properties of the constituent powders are considered. The average particle size of Al powders (D50 ≈ 43.3 µm) is approximately one order of magnitude larger than that of SiC reinforcement particles (D50 ≈ 5.08 µm). This disparity in particle size allows for the good dispersion of hard SiC particles within the Al matrix after sintering, resulting in increased interfacial area between matrix and reinforcement, further limiting dislocation motion by solid particle strengthening and/or possible grain refinement effects. As a result, a pronounced dispersion-strengthening mechanism, commonly associated with the Orowan mechanism^41^, is expected to contribute to the observed hardness enhancement.

Consistent with the present results, several studies have reported significant increases in Vickers microhardness with increasing SiC content in powder-metallurgy-processed Al/SiC composites, with this effect being particularly pronounced when fine SiC particles are used as reinforcement^42^. In this context, the approximately 34% hardness increase measured for the composite containing 10 vol.% SiC is in good agreement with trends reported in the literature and represents an expected level considering the employed particle sizes and sintering conditions. Studies reported in the literature on Al/SiC microparticle composites fabricated by powder metallurgy support the observed trend in the present work. Selvakumar et al. investigated powder metallurgy composites based on an Al–4 wt% Cu matrix reinforced with 0–15 wt% SiC and reported that increasing SiC content led to pronounced improvements in microhardness and compressive strength, while simultaneously causing a reduction in thermal conductivity^43^. Similarly, Abu-Oqail et al. demonstrated that increasing the SiC particle content in hot-pressed Al–xSiC composites enhanced microhardness and tribological performance, which was attributed to the homogeneous distribution of reinforcement particles and the formation of a strong matrix–reinforcement interface^44^. In another study, Al-Taa’y et al. reported that in powder-metallurgy-processed Al/SiC composites, an increase in the volume fraction of SiC particles with sizes ranging from 20 to 45 µm resulted in clear improvements in hardness and mechanical strength, despite a slight decrease in density^45^. When these studies are considered collectively, it can be concluded that the approximately 34% increase in hardness observed in the present study when transitioning from pure Al to the composite reinforced with 10 vol.% SiC falls well within the typical behaviour range of powder-metallurgy-fabricated Al/SiC microparticle composites. Although increasing SiC content results in higher porosity and lower relative density, the associated local stiffness inhomogeneities may promote micro-deflection and vibration during drilling, potentially affecting dimensional accuracy (DD and CD). However, the significant increase in hardness and reduction in plastic deformation caused by SiC reinforcement in the matrix contribute to a more stable cutting process; this prevents excessive increases in thrust force at low feed rates and reduces negative impacts on hole quality.

### Drilling of Al/SiC composites

In this research, drilling was chosen due to its crucial importance in the assembly process of AMC components used in industrial applications. Al/SiC composites are generally joined using mechanical fastening techniques such as bolts, rivets, and screws, and drilling holes with high surface and geometric accuracy is required to ensure a proper fit, efficient load transfer, and increased fatigue strength. Therefore, improving the quality of holes formed during the drilling process is a critical requirement for the safe and trouble-free integration of Al/SiC composite parts into structural systems.

#### S/N analysis

S/N ratio analysis was employed to evaluate the deviation of the experimental results from the desired target values and to determine the optimal drilling parameters. In order to achieve high hole quality, the minimization of Fz, Ra, DD, and CD was targeted. Accordingly, the smaller-is-better quality characteristic was selected for the S/N analysis, and the S/N ratios were calculated using Eq. (3). Experimental results, Standard Deviation (SD) values, and calculated S/N ratios are presented in Table 3. The experimental mean values and the calculated mean S/N ratios of the responses were determined as 588.111 N and − 54.765 dB for Fz, 5.923 µm and − 15.212 dB for Ra, 0.086 mm and 21.563 dB for DD, and 0.032 mm and 30.245 dB for CD, respectively.

**Table 3:** Responses, standard deviations and calculated S/N values.

Exp.No	Vc (m/min)	f (mm/rev)	PA (°)	RR (%)	Fz (N)	SD	Fz-S/N (dB)	Ra (µm)	SD	Ra-S/N (dB)	Average hole diameter	DD (mm)	SD	DD-S/N (dB)	CD (mm)	SD	CD-S/N (dB)
1	25	0.08	100	0	653	15.28	− 56.298	6.578	0.282	− 16.362	5.079	0.079	0.0045	22.047	0.033	0.0021	29.630
2	25	0.08	118	5	351	11.53	− 50.906	5.130	0.240	− 14.202	5.058	0.058	0.0035	24.731	0.025	0.0017	32.041
3	25	0.08	136	10	214	8.50	− 46.608	4.050	0.270	− 12.149	5.049	0.049	0.0030	26.196	0.017	0.0015	35.391
4	25	0.16	100	0	914	26.76	− 59.219	7.873	0.345	− 17.923	5.086	0.086	0.0050	21.310	0.037	0.0032	28.636
5	25	0.16	118	5	571	18.52	− 55.133	6.714	0.260	− 16.540	5.075	0.075	0.0042	22.499	0.027	0.0025	31.373
6	25	0.16	136	10	394	14.73	− 51.910	5.290	0.265	− 14.469	5.063	0.063	0.0040	24.013	0.020	0.0017	33.979
7	25	0.24	100	5	972	28.50	− 59.753	7.507	0.294	− 17.509	5.094	0.094	0.0055	20.537	0.036	0.0031	28.874
8	25	0.24	118	10	664	19.08	− 56.443	6.758	0.235	− 16.596	5.079	0.079	0.0046	22.047	0.028	0.0020	31.057
9	25	0.24	136	0	717	23.18	− 57.110	7.787	0.270	− 17.827	5.102	0.102	0.0055	19.828	0.032	0.0021	29.897
10	50	0.08	100	10	400	12.53	− 52.041	3.414	0.265	− 10.665	5.073	0.073	0.0040	22.734	0.029	0.0025	30.752
11	50	0.08	118	0	430	12.01	− 52.669	5.123	0.155	− 14.190	5.095	0.095	0.0046	20.446	0.034	0.0030	29.370
12	50	0.08	136	5	300	10.50	− 49.542	3.809	0.170	− 11.616	5.079	0.079	0.0045	22.047	0.025	0.0020	32.041
13	50	0.16	100	5	699	19.86	− 56.890	5.831	0.282	− 15.315	5.093	0.093	0.0050	20.630	0.037	0.0031	28.636
14	50	0.16	118	10	514	16.52	− 54.219	4.710	0.198	− 13.460	5.081	0.081	0.0040	21.830	0.029	0.0025	30.752
15	50	0.16	136	0	575	17.79	− 55.193	6.011	0.295	− 15.579	5.103	0.103	0.0060	19.743	0.036	0.0031	28.874
16	50	0.24	100	10	805	23.54	− 58.116	6.243	0.224	− 15.908	5.103	0.103	0.0067	19.743	0.040	0.0032	27.959
17	50	0.24	118	0	825	22.52	− 58.329	7.344	0.220	− 17.319	5.121	0.121	0.0070	18.344	0.046	0.0035	26.745
18	50	0.24	136	5	588	19.14	− 55.388	6.449	0.232	− 16.190	5.107	0.107	0.0065	19.412	0.038	0.0025	28.404
Average					588.111	17.81	− 54.765	5.923	0.250	− 15.212	5.086	0.086	0.0049	21.563	0.032	0.0025	30.245
The largest					972	28.50	− 46.608	7.873	0.345	− 10.665	5.121	0.121	0.0070	26.196	0.046	0.0035	35.391
The smallest					214	8.50	− 59.753	3.414	0.155	− 17.923	5.049	0.049	0.0030	18.344	0.017	0.0015	26.745

**Fig. 8:**
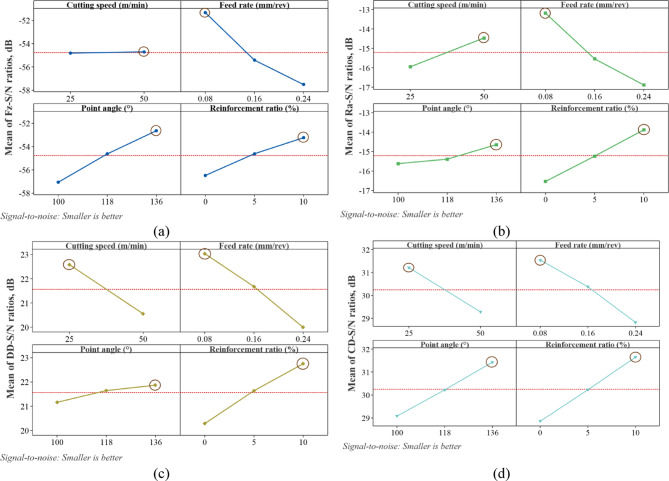
Main effects plot for S/N ratios: (**a**) Fz, (**b**) Ra, (**c**) DD, (**d**) CD.

The calculated mean response values corresponding to each level of the control factors used in the drilling process are presented in Table 4. In addition, the main effects plots of the S/N ratios for the responses are shown in Fig. 8. Since the improvement of hole quality requires the minimization of all response variables, the levels of the control factors yielding the lowest mean response values indicate the optimal experimental conditions for each response. Similarly, the highest levels of the control factors observed in the main effects plots of the S/N ratios also represent the optimal experimental conditions. Based on the combined evaluation of Table 4 and Fig. 7, the optimal drilling condition for Fz and Ra was determined as $Vc_{2} f_{1} PA_{3} RR_{3}$ (Vc: 50 m/min, f: 0.08 mm/rev, PA: 136°, and RR: 10%), whereas the optimal condition for DD and CD was identified as $Vc_{1} f_{1} PA_{3} RR_{3}$ (Vc: 25 m/min, f: 0.08 mm/rev, PA: 136°, and RR: 10%).

**Table 4:** Response table for the mean values of Fz, Ra, DD, and CD.

Level	Fz (N)				Ra (µm)			
	Vc	f	PA	RR	Vc	f	PA	RR
1	605.6	391.3*	740.5	685.7	6.410	4.684*	6.241	6.786
2	570.7*	611.2	559.2	580.2	5.437*	6.072	5.963	5.907
3	–	761.8	464.7*	498.5*		7.015	5.566*	5.078*
Delta	34.9	370.5	275.8	187.2	0.973	2.331	0.675	1.708
Overall average Fz = 588.111 N					Overall average Ra = 5.923 µm			

Level	DD (mm)				CD (mm)			
	Vc	f	PA	RR	Vc	f	PA	RR
1	0.0761*	0.0722*	0.0880	0.0977	0.0283*	0.0272*	0.0353	0.0363
2	0.0950	0.0835	0.0848	0.0843	0.0349	0.0310	0.0315	0.0313
3		0.1010	0.0838*	0.0747*		0.0367	0.0280*	0.0272*
Delta	0.0189	0.0288	0.0042	0.0230	0.0066	0.0095	0.0073	0.0092
Overall average DD = 0.0856 mm					Overall average CD = 0.0316 mm			

*Optimal levels of control factors

#### Evaluation of 3D plots

**Fig. 9:**
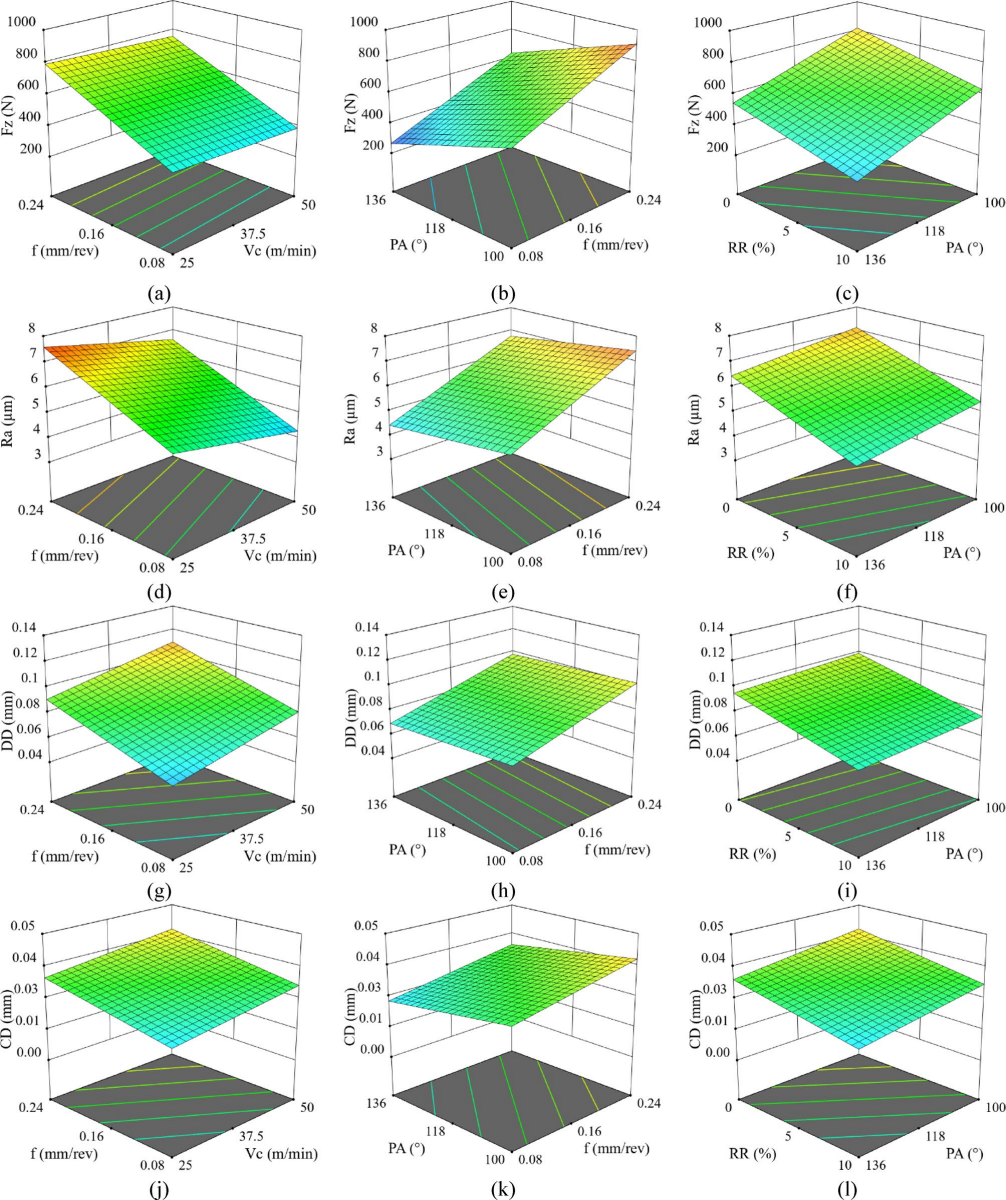
Three-dimensional plots of the responses: (**a**–**c**) for Fz, (**d**–**f**) for Ra, (**g**–**i**) for DD, and (j–l) for CD.

The three-dimensional response surfaces presented in Fig. 9 illustrate the combined effects of the drilling parameters on responses. As illustrated in Fig. 9a–c, the Fz increases markedly with increasing feed rate, indicating that feed rate is the dominant parameter governing the axial cutting load during drilling. This behaviour is primarily attributed to the increase in uncut chip thickness and the higher material removal per revolution, which directly intensify the resistance encountered by the drill. The pronounced influence of feed rate observed in the drilling of DHP Al/SiC composites is in good agreement with existing literature^46,47^, where feed rate is consistently reported as the most critical factor affecting Fz in particle-reinforced AMCs. Although Vc, PA, and RR also influence the cutting mechanics, their effects remain secondary compared to the dominant contribution of feed rate to the overall cutting load. A moderate reduction in the Fz of the cutting process is observed supplied with an increase in trimming speed, which could be well understood as better cutting efficacy in addition to decreased adhesion at the workpiece-tool interface. On a similar note, larger PA and a greater RR typically lead to higher Fz, which actually favors better cutting behavior and less material smearing. A similar trend is observed for Ra in Fig. 9d–f. Ra increases significantly with feed rate, whereas it shows the opposite behavior with Vc, where higher Vc lead to improved surface finish. The use of a higher PA and RR to suppress Ra values indicates improved tool engagement and increased composite stiffness, which together help maintain better surface quality and enhance drilling performance. This outcome is consistent with the findings reported by Kumaran et al.^48^, who demonstrated that higher PA in Al/SiC composites reduce Fz, thereby leading to lower Ra values and improved tool–workpiece interaction. Furthermore, an increased RR enhances the stiffness of the composite, contributing to the preservation of surface integrity during drilling. Nevertheless, the inherently abrasive nature of the reinforcements may accelerate tool wear, emphasizing the importance of careful optimization of drilling parameters.

In contrast, the response surfaces for DD and CD in Fig. 9g–l reveal a stronger sensitivity to feed rate and a different influence of Vc. Both DD and CD increase substantially with increasing feed rate, reflecting the adverse effects of higher cutting loads on dimensional and form accuracy. Unlike Fz and Ra, higher Vc tend to slightly deteriorate DD and CD, which may be associated with increased dynamic effects, thermal expansion, or tool deflection at elevated speeds. Conversely, larger PA and higher RR consistently reduce both DD and CD, indicating improved hole stability and resistance to geometric distortion. Overall, the 3D plots confirm that low feed rate, high PA, and higher RR are favorable for achieving superior hole quality, while the effect of Vc depends on whether surface integrity or geometric accuracy is prioritized. At higher cutting speeds, the deterioration in hole circularity and cylindricity is mainly associated with increased heat generation, tool deflection, and dynamic machining effects^49^. Elevated cutting speeds increase the amount of heat generated at the tool–workpiece interface during drilling, resulting in significant thermal loading of both the drill and the workpiece material. Bono et al. demonstrated that this thermal input causes radial thermal expansion of the workpiece during machining, followed by contraction upon cooling to room temperature, which in turn leads to measurable deviations in hole diameter and cylindricity due to thermally induced geometric distortion^50^. Moreover, higher cutting speeds promote dynamic cutting forces and vibration, resulting in elastic deflection and run-out of the HSS drill, especially in the presence of abrasive SiC particles. These combined thermal and dynamic effects produce eccentric cutting paths and consequently increase circularity and cylindricity errors.

**Fig. 10:**
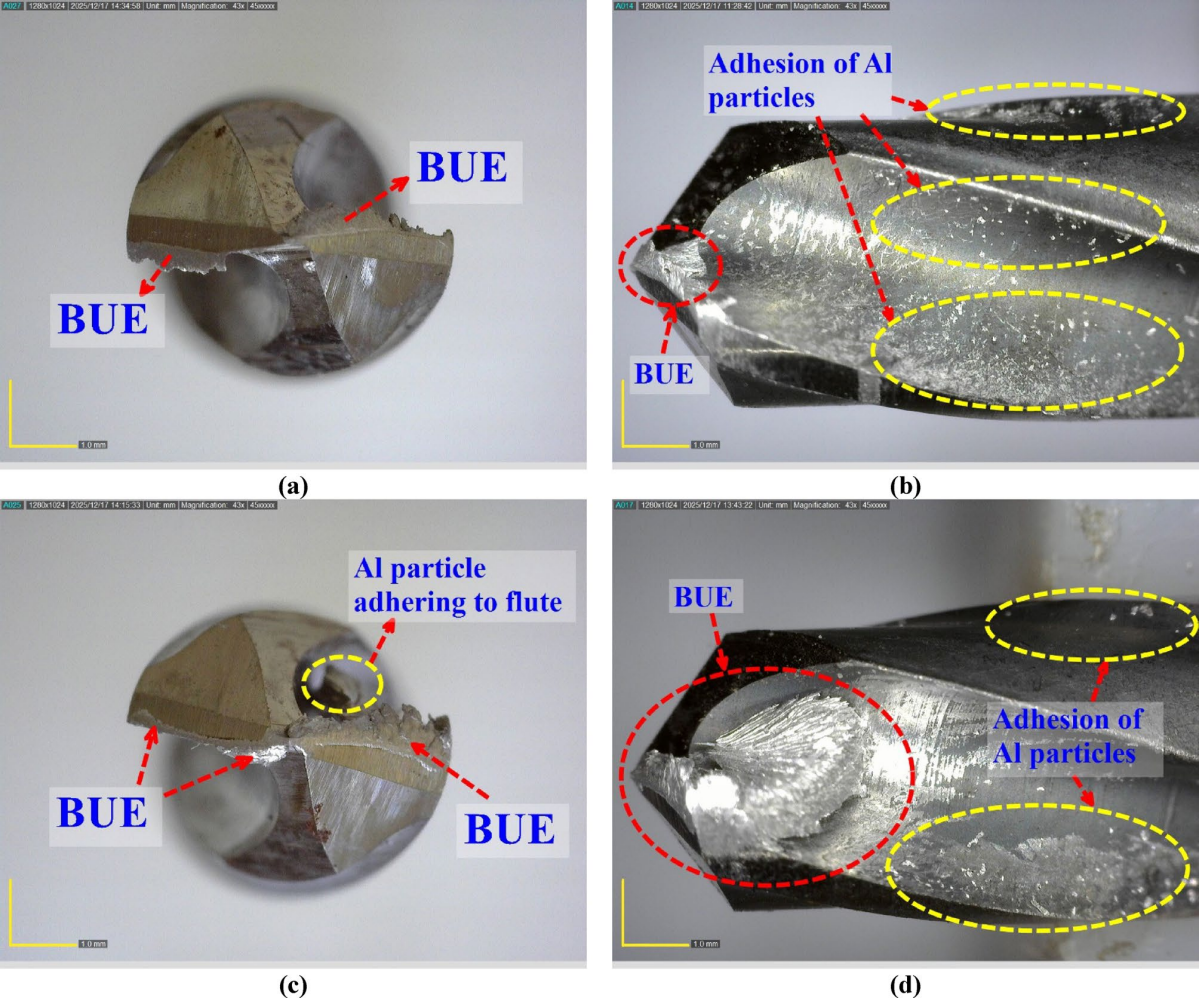
Top and side views of the drills after the drilling process; 3rd test condition (**a**, **b**), 9th test condition (**c**, **d**).

Tool conditions after drilling within the 3rd experimental condition (lowest feed rate and high RR) and the 9th experimental condition (highest feed rate and the lowest RR, that is, 0% of SiC) are compared in Fig. 10. Adherence of the Al, such as severe and extensive BUE formation, particularly at the flank and the cutting edges, under the 9th condition, must be ascribed to high feed rates, resulting in the complete ductile, unreinforced Al matrix promoting adhesive wear in the process. In contrast, lowest feed rates reduce the cutting loads and tribological interactions to foster limited BUE formation and un-reactive adhesion of the Al with the least activity at the back of the flutes, though both conditions have hard reinforcement grains packed against the metallic matrix. In this case, the initial concern regarding formaldehyde formation is considerably more severe compared to the control. However, examination of the images and the observed cutting conditions eliminates these concerns: reducing the feed rate and, more importantly, increasing the reinforcement content result in stable cutting behavior and minimal material adhesion.

**Fig. 11:**
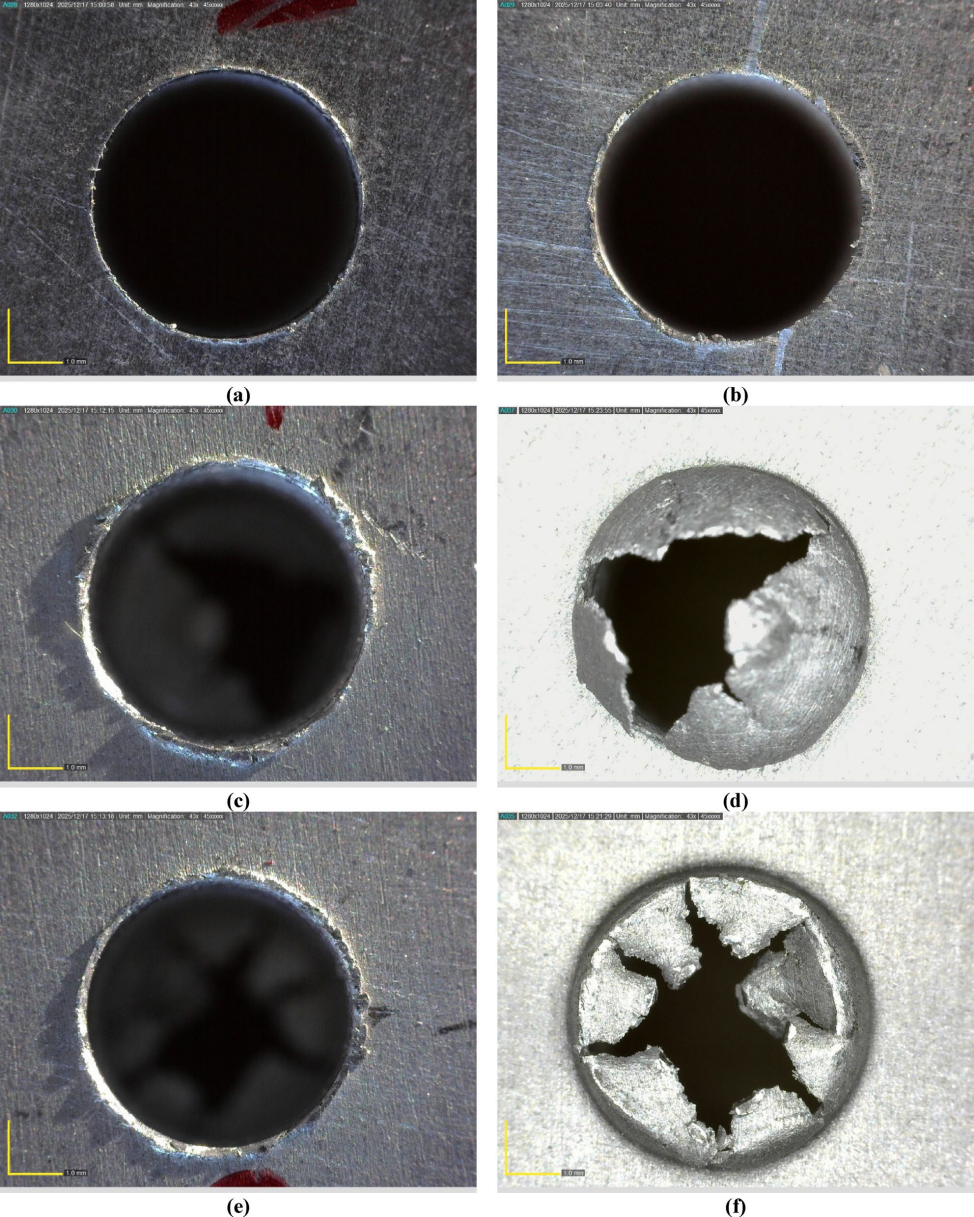
Front and rear holes obtained under different experimental conditions: (**a**, **b**) 3rd experiment, (**c**, **d**) 11th experiment, and (**e**, **f**) 17th experiment.

Figure 11 compares the entrance and exit hole morphologies obtained under different drilling conditions: (a–b) the 3rd experiment, (c–d) the 11th experiment, and (e–f) the 17th experiment. The 3rd experimental condition, characterized by the lowest feed rate and the highest RR, exhibits a clean hole exit with negligible burr formation, which can be attributed to the increased hardness and reduced plastic deformation resulting from the presence of hard SiC ceramic particles. The higher reinforcement content enhances the stiffness of the composite and promotes a more brittle cutting response, which effectively suppresses material smearing and plastic flow at the hole exit. This behaviour is consistent with findings reported in the existing literature. Basar et al. demonstrated that increasing the SiC content in AMCs leads to a significant increase in hardness while reducing transverse rupture strength, indicating a transition toward a stiffer and more brittle material response during machining^13^. Similarly, Aurich et al. reported that higher ceramic reinforcement fractions improve resistance to plastic deformation and reduce adhesive material flow at the tool–workpiece interface^51^. The incorporation of hard SiC particles restricts dislocation motion in the Al matrix, enhances load-bearing capacity, and limits excessive ductile deformation, thereby improving dimensional stability and reducing burr formation during drilling. These literature-supported observations are in good agreement with the present results, confirming that increased SiC content plays a critical role in improving hole exit quality by promoting a harder and less ductile cutting behaviour. As opposed to the 11th trial with the highest Vc, the lowest feed rate, and the lowest RR (pure Al), burrs were distinctly present at the exit side due to the extreme smearing and adhesive characteristics of the Al’s ductile matrix. This effect became even more apparent in the 17th condition with the highest Vc and highest feed rate under zero reinforcement, inflicting severe exit damage, irregular edge deformation, and massive burr formation on the surface. Taken together, this figure shows that burring is enhanced in the case of low magnitudes of reinforcement material due to the ductile nature of pure Al, while an increase in the SiC content enhances the hardness of the composite and fosters a less ductile, more brittle response, which in turn diminishes plastic flow and burr growth at the hole exit.

**Fig. 12:**
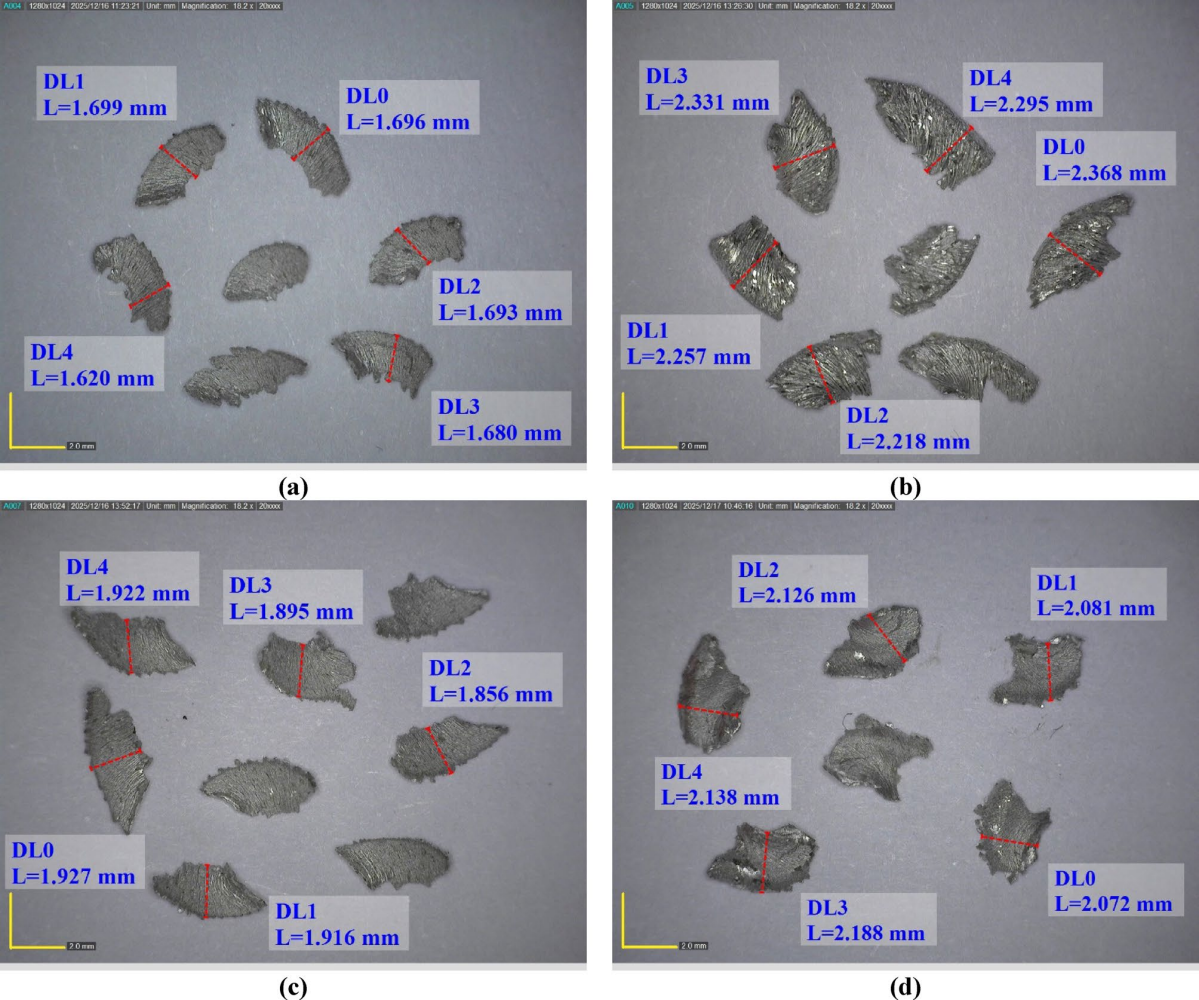
Chip images according to experimental conditions: (**a**) 3rd, (**b**) 9th, (**c**) 10th, and (**d**) 16th.

Chips formed in varied conditions are accounted for as per the chip morphologies presented in Fig. 12, proving the combined effect of feed rate, Vc, and RR on the chip formations. For the 3rd experiment carried out at the lowest feed rate and highest RR, the chips were short, fragmented, and irregular, meaning the cut was more brittle owing to high SiC content and low chip load. This suggests that the presence of hard particles and low feed in the cutting zone has restricted the continuous plastic flow, thus encouraging theoretical chip breakage. Conversely, the 9th experiment carried out at a very high feed rate and RR (0% SiC) produces longer and continuously flowing chips in shape, thus reflecting pure Al cut under very high chip thickness conditions tending towards ductile flow and smearing. Whereas the 10th experiment, combining processing at the highest Vc with both the lowest feed rate and highest RR, yields moderately sized but still discontinuous chips, hinting that higher cutting speed weakens chip segmentation at certain levels, whereas the high reinforcement promotes brittle response. Last but not least, in the 16th experiment with the greatest Vc, feed rate, and RR, the chips are relatively large, more or less fractured, suggesting an interference between higher chip load (favoring continuity of chip) and high SiC content plus Vc (favoring chip rupture). The overall lesson garnered from the figure is that, in essence, low feed with high RR promotes fragmented, brittle chips, while high feed and low RR encourage flow of continuous, ductile seating. This observation is in good agreement with the findings reported by Salur et al.^52^, who showed that drilling of direct hot-pressed Al/SiC composites at low feed rates and high RR leads to the formation of fragmented, brittle chips, whereas higher feed rates combined with lower RR favor the development of continuous, ductile chips.

#### ANOVA results

Table 5 summarizes the ANOVA results for the Fz, Ra, DD, and CD responses at a 95% confidence level. For Fz, the analysis clearly indicates that feed rate is the most influential parameter (F = 136.20, *p* < 0.001), accounting for 53.49% of the total variance, followed by PA (30.28%) and RR (13.57%), both of which are statistically significant (*p* < 0.001). Vc does not exhibit a statistically significant effect on Fz within the investigated range (*p* = 0.088), contributing only 0.70% to the total variation. A similar trend is observed for Ra, where feed rate again dominates the response with the highest contribution (52.58%, *p* < 0.001). RR (27.93%) and Vc (13.57%) also show significant effects on Ra, whereas PA has a relatively minor influence (4.40%). The high coefficients of determination (R^2^ > 98% for both Fz and Ra) and low error contributions confirm the robustness and adequacy of the developed statistical models.

**Table 5:** ANOVA results of the responses.

Factors	DF	Seq SS	Adj MS	F-value	*p*-Value	PCR %
Fz (N)						
Vc	1	5478	5478	3.58	0.088	0.70
f	2	416,595	208,297	136.20	*p* < 0.001	53.49
PA	2	235,792	117,896	77.09	*p* < 0.001	30.28
RR	2	105,662	52,831	34.55	*p* < 0.001	13.57
Error	10	15,293	1529			1.96
Total	17	778,820				100
R^2^ : %98.04, R^2^ (adj): %96.66, R^2^ (pred): %93.64						
Ra (µm)						
Vc	1	4.2564	4.25639	89.57	*p* < 0.001	13.57
f	2	16.4935	8.24673	173.53	*p* < 0.001	52.58
PA	2	1.3811	0.69056	14.53	0.001	4.40
RR	2	8.7594	4.37972	92.16	*p* < 0.001	27.93
Error	10	0.4752	0.04752			1.52
Total	17	31.3656				100
R^2^ : %98.48, R^2^ (adj): %97.42, R^2^ (pred): %95.09						
DD (mm)						
Vc	1	0.001606	0.001606	405.90	*p* < 0.001	27.52
f	2	0.002532	0.001266	320.07	*p* < 0.001	43.40
PA	2	0.000057	0.000028	7.18	0.012	0.97
RR	2	0.001600	0.000800	202.30	*p* < 0.001	27.43
Error	10	0.000040	0.000004			0.68
Total	17	0.005834				100
R^2^ : %99.32, R^2^ (adj): %98.85, R^2^ (pred): %97.80						
CD (mm)						
Vc	1	0.000193	0.000193	78.76	*p* < 0.001	21.34
f	2	0.000274	0.000137	55.81	*p* < 0.001	30.25
PA	2	0.000161	0.000081	32.87	*p* < 0.001	17.81
RR	2	0.000253	0.000126	51.47	*p* < 0.001	27.89
Error	10	0.000025	0.000002			2.71
Total	17	0.000906				100
R^2^ : %97.29, R^2^ (adj): %95.39, R^2^ (pred): %91.22						

**Fig. 13:**
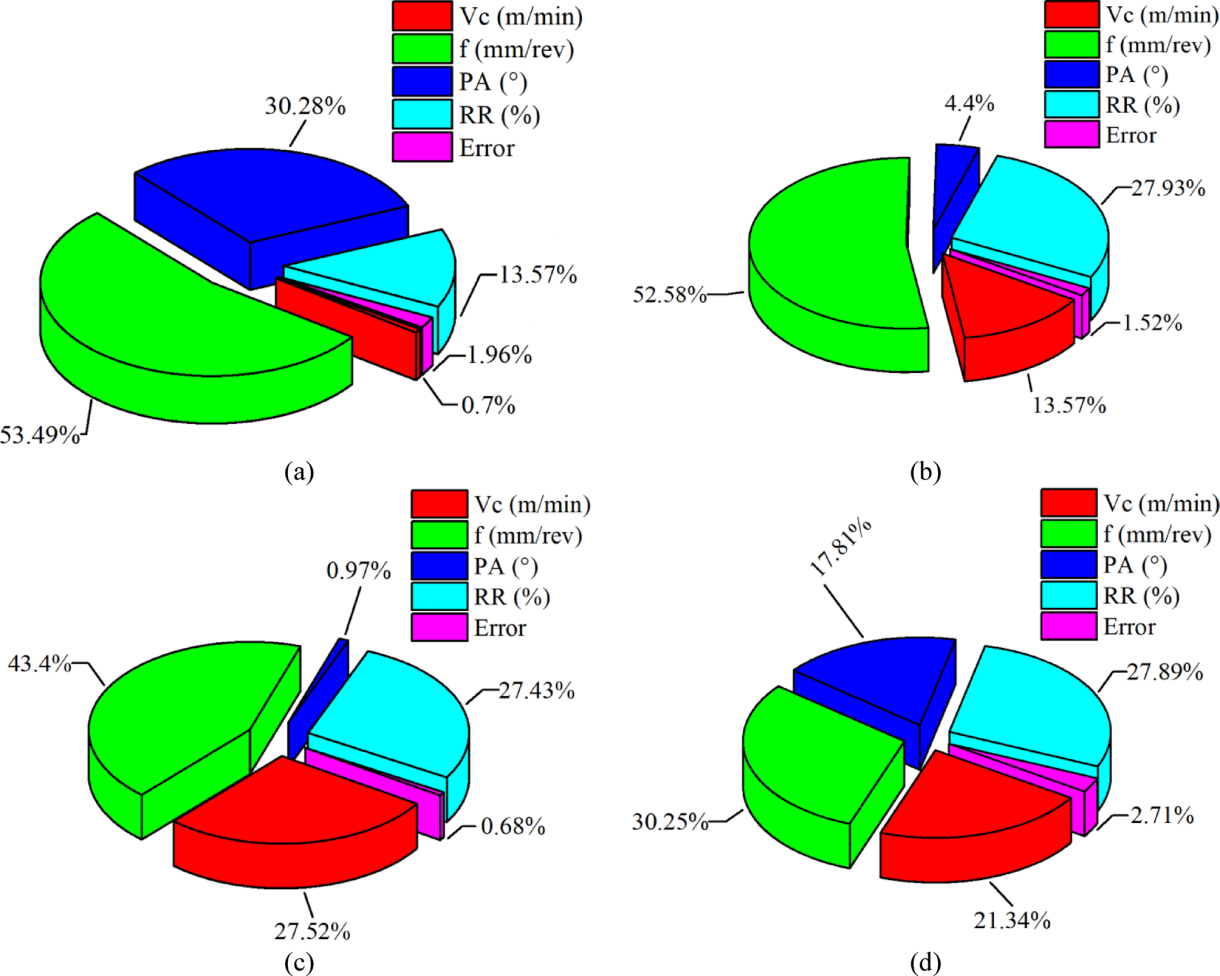
Contribution ratios of the control factors on the responses: (**a**) Fz, (**b**) Ra, (**c**) DD, (**d**) CD.

Controlling the geometrical hole quality characteristics of DD and CD, feed rate in DD is the main factor, the totals amounted to 43.40% and 30.25% of the variance, respectively (*p* < 0.001). Unlike Fz, speed and reinforcement influence DD fairly equally and significantly, with share ratios of 27.52% and 27.43%, such that it may be seen that speed-related effects together with stiffness of the material play a major role in deciding dimension accuracy. PA was found to be statistically significant and contributed a similar proportion to DD (0.97%). In contrast, for the case of CD, all factors come out statistically significant with the RR (27.89%), Vc (21.34%), and PA (17.81%) being the major contributors collectively to hole form accuracy. Very high R^2^, high enough to be declared as reliable, in DD (99.32%) and CD (97.29%) seems to prove beyond doubt that the proposed models are capable of representing the primary mechanisms behind hole geometry. The ANOVA results clearly quantify the relative influence of the drilling parameters and establish a numerical hierarchy for process control. Feed rate was the dominant factor affecting Fz (53.49%), Ra (52.58%), DD (43.40%), and CD (30.25%). Point angle was identified as the second most influential parameter for Fz (30.28%), whereas the reinforcement ratio ranked second for Ra (27.93%), DD (27.43%), and CD (27.89%). Conversely, cutting speed exhibited the lowest contribution to Fz (0.70%), while point angle showed the lowest contribution to Ra (4.40%), DD (0.97%), and CD (17.81%). Figure 13 illustrates the percentage contribution of the control factors on each response, clearly demonstrating that feed rate is the dominant parameter for all hole quality characteristics, while RR, Vc, and PA exhibit varying levels of influence depending on the response considered.

#### Regression analysis

Regression analysis was employed to determine the cause–effect relationships between the control factors and the responses measured after the drilling of the Al/SiC composites. First-order regression equations were developed for responses, and these models are presented in Eqs. (4)–(7), respectively.

4 $$Fz \left( N \right) = 1267.6 - 1.396Vc + 2316f - 7.662PA - 18.72RR$$

5 $$Ra \left( {\mu m} \right) = 8.118 - 0.03890Vc + 14.567f - 0.01875PA - 0.1709RR$$

6 $$DD \left( {mm} \right) = 0.05355 + 0.000756Vc + 0.18021f - 0.000116PA - 0.0023RR$$

7 $$CD \left( {mm} \right) = 0.04090 + 0.000262Vc + 0.05937f - 0.000204PA - 0.000917RR$$

According to the regression analysis results, the coefficients of determination for the mathematical model developed for Fz were obtained as R^2^ = 96.38%, adjusted R^2^ = 95.27%, and predicted R^2^ = 93.30%. For the Ra model, the corresponding values were R^2^ = 97.80%, adjusted R^2^ = 97.13%, and predicted R^2^ = 95.59%. Similarly, the regression model developed for DD yielded coefficients of determination of R^2^ = 98.36%, adjusted R^2^ = 97.85%, and predicted R^2^ = 96.82%, whereas the model for CD resulted in R^2^ = 96.83%, adjusted R^2^ = 95.86%, and predicted R^2^ = 93.73%. These results indicate that the regression equations developed for all responses exhibit high predictive capability. Moreover, the strong agreement between the experimental and predicted values suggests that the relationships between the control factors and the responses are statistically significant. In general, a coefficient of determination (R^2^) greater than 80% is considered indicative of a statistically meaningful and reliable regression model^53^, which further confirms the adequacy of the developed models in the present study.

**Fig. 14:**
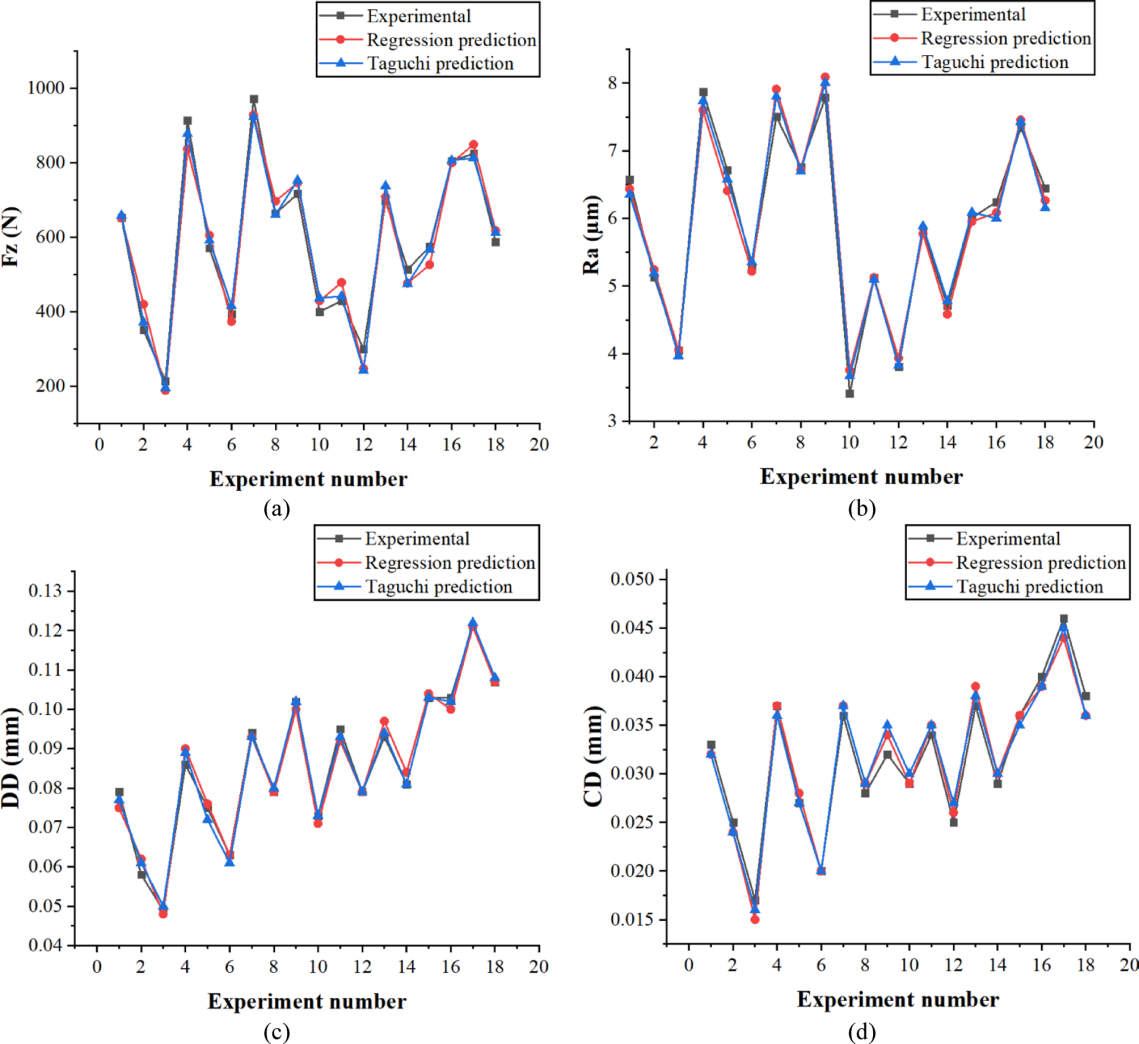
Experimental and predicted results for the responses: (**a**) Fz, (**b**) Ra, (**c**) DD, and (**d**) CD.

A comparison between the experimental data and the calculated predictions for Taguchi and regression models is provided in Fig. 14. The prediction performance of the models had been assessed by obtaining the Mean Absolute Percentage Error (MAPE). From the analysis results, Taguchi’s method has a MAPE of 5.03 for the predictions of Fz, while the regression one has a MAPE of 7.05 for the same prediction variable predictions. The Taguchi method showed even lower MAPE in Ra and DD by 2.28 and 1.59 compared with the regression model with 2.70 and 2.27 correspondingly. In case of CD, comparing both methods, their predictive power and results were almost expected. The MAPE were a close match as they read 3.59 for the Taguchi method and 3.50 for the regression model. The low MAPE on all response variables shows that both methods predict well and are in good agreement with the experimental data.

#### Confirmation test

In the final stage of the Taguchi method, confirmation tests are conducted to verify whether the improvement predicted by the selected optimal control factors and their corresponding levels can be achieved in practice. According to the Taguchi analysis, the optimal combination of control factors was determined as $Vc_{2} f_{1} PA_{3} RR_{3}$ for Fz and Ra, whereas the optimal condition for DD and CD was identified as $Vc_{1} f_{1} PA_{3} RR_{3}$. The predicted optimal values of Fz, Ra, DD, and CD were calculated using Eqs. (8)–(11), respectively. In these calculations, the response table for the mean values presented in Table 4 was employed.

8 $$Fz_{opt} = \left( {Vc_{2} - T_{Fz} } \right) + \left( {f_{1} - T_{Fz} } \right) + \left( {PA_{3} - T_{Fz} } \right) + \left( {RR_{3} - T_{Fz} } \right) + T_{Fz}$$

9 $$Ra_{opt} = \left( {Vc_{2} - T_{Ra} } \right) + \left( {f_{1} - T_{Ra} } \right) + \left( {PA_{3} - T_{Ra} } \right) + \left( {RR_{3} - T_{Ra} } \right) + T_{Ra}$$

10 $$DD_{opt} = \left( {Vc_{1} - T_{DD} } \right) + \left( {f_{1} - T_{DD} } \right) + \left( {PA_{3} - T_{DD} } \right) + \left( {RR_{3} - T_{DD} } \right) + T_{DD}$$

11 $$CD_{opt} = \left( {Vc_{1} - T_{CD} } \right) + \left( {f_{1} - T_{CD} } \right) + \left( {PA_{3} - T_{CD} } \right) + \left( {RR_{3} - T_{CD} } \right) + T_{CD}$$

The mean values of the responses obtained from the experimental study are represented by $T_{Fz}$ for Fz, $T_{Ra}$ for Ra, $T_{DD}$ for DD, and $T_{CD}$ for CD, while the predicted optimum response values are denoted by $Fz_{opt}$, $Ra_{opt}$, $DD_{opt}$, and $CD_{opt}$, respectively. The predicted optimum values of Fz, Ra, DD, and CD were calculated as 160.867 N, 2.996 µm, 0.050 mm, and 0.016 mm, respectively. The confirmation test results for the responses are presented in Table 6. In the confirmation experiment, the measured Fz and Ra values were 170 N and 3.250 µm, respectively, whereas the values predicted by the Taguchi method were 160.867 N and 2.996 µm. In addition, when the results obtained under the optimum conditions were compared with those of the initial parameter setting, the improvements in the S/N ratios for Fz and Ra were 11.689 dB and 6.124 dB, respectively, corresponding to improvement rates of 20.76% and 37.43%. No additional confirmation experiments were conducted for DD and CD. Instead, the results of Experiment 3, which is included in the Taguchi L_18_ experimental design, were used. For DD and CD, the confirmation test results were 0.049 mm and 0.017 mm, respectively, while the corresponding values predicted by the Taguchi method were 0.050 mm and 0.016 mm. Moreover, compared with the initial parameter setting, the improvements in the S/N ratios for DD and CD under the optimum conditions were 4.149 dB and 5.761 dB, respectively, corresponding to improvement rates of 18.82% and 19.44%. Overall, the confirmation test results of the hole quality indicators demonstrate that the optimization procedure based on the Taguchi method was successfully implemented.

**Table 6:** Confirmation test results for the responses.

Taguchi optimization	Initial parameter	Optimal control factors and levels	
		Prediction	Experimental
Fz (N)			
	$Vc_{1} f_{1} PA_{1} RR_{1}$	$Vc_{2} f_{1} PA_{3} RR_{3}$	
Fz (N)	653	160.867	170
Fz-S/N ratio (dB)	− 56.298	− 44.129	− 44.609
Percentage improvement in S/N ratio = %20.76			
Ra (µm)			
	$Vc_{1} f_{1} PA_{1} RR_{1}$	$Vc_{2} f_{1} PA_{3} RR_{3}$	
Ra (µm)	6.578	2.996	3.250
Ra-S/N ratio (dB)	− 16.362	− 9.531	− 10.238
Percentage improvement in S/N ratio = %37.43			
DD (mm)			
	$Vc_{1} f_{1} PA_{1} RR_{1}$	$Vc_{1} f_{1} PA_{3} RR_{3}$	
DD (mm)	0.079	0.050	0.049
DD-S/N ratio (dB)	22.047	26.021	26.196
Percentage improvement in S/N ratio = %18.82			
CD (mm)			
	$Vc_{1} f_{1} PA_{1} RR_{1}$	$Vc_{1} f_{1} PA_{3} RR_{3}$	
CD (mm)	0.033	0.016	0.017
CD-S/N ratio (dB)	29.630	35.918	35.391
Percentage improvement in S/N ratio = %19.44			

The experimental responses obtained under the optimum drilling conditions and the values predicted by the estimation models are presented in Table 7. The Absolute Percentage Error (APE) between the experimental results obtained under the optimum conditions and the Taguchi-predicted values was calculated as 5.37 for Fz, 7.82 for Ra, 2.04 for DD, and 5.88 for CD. In comparison, the APE between the experimental results and the first-order regression–predicted values was calculated as 9.46 for Fz, 5.23 for Ra, 2.04 for DD, and 11.76 for CD. These results suggest that the predictive capability of both methods is much higher. However, in general, the Taguchi method produces a little better accuracy. The relatively low APE also indicates that the forementioned use of the Taguchi method in optimization is effective. This finding is consistent with the results reported by Basar et al.^13^, who demonstrated that the Taguchi method is a reliable and robust approach for optimizing drilling parameters in a wide range of materials, including Al/SiC composites. Their study showed that the method effectively reduces experimental errors, improves quality characteristics, and ensures consistent and reproducible outcomes through systematic statistical analysis and confirmation experiments. The low APE observed in both studies therefore highlights the robustness and reliability of the Taguchi method in drilling process optimization.

**Table 7:** Comparison of the experimental and predicted values of the responses under optimum conditions.

Responses	Experimental result	Taguchi prediction	Absolute error %	Regression prediction	Absolute error %
Fz (N)	170	160.867	5.37	153.917	9.46
Ra (µm)	3.250	2.996	7.82	3.080	5.23
DD (mm)	0.049	0.050	2.04	0.048	2.04
CD (mm)	0.017	0.016	5.88	0.015	11.76

#### Calculation of the confidence interval

To evaluate the reliability of the optimization process performed using the Taguchi method, the confidence intervals (CI) of the predicted responses were calculated using Eqs. (12) and (13)^54,55^. The various parameter values used in the calculations are given in Table 8.

12 $$CI = \sqrt {F_{{\ddot{\alpha }:1,{\mathrm{Sd}}_{{\mathrm{e}}} }} \overline{V}_{e} \left( {\frac{1}{{n_{eff} }} + \frac{1}{{\overline{r}}}} \right)}$$

13 $$n_{eff} = \frac{n}{{1 + T_{dof} }}$$

**Table 8:** Various parameters and their values used in the calculation of the CI of the responses.

No	1	2	3	4	5	6	7	8
Sembol	$F_{{\ddot{\alpha }:1,{\mathrm{Sd}}_{{\mathrm{e}}} }}$	$\ddot{\alpha }$	${\mathrm{Sd}}_{{\mathrm{e}}}$	$\overline{{V_{e} }}$	$\overline{r}$	$n_{eff}$	$n$	$T_{dof}$
Fz	4.9646	0.05	10	1529	3	2.25	18	7
Ra	4.9646	0.05	10	0.04752	3	2.25	18	7
DD	4.9646	0.05	10	0.000004	3	2.25	18	7
CD	4.9646	0.05	10	0.000002	3	2.25	18	7

In the calculation of the CI for the responses, Eqs. (14)–(17) were used, respectively. The CI were calculated as ± 76.83 N for Fz, ± 0.428 µm for Ra, ± 0.0039 mm for DD, and ± 0.0028 mm for CD.

14 $$\left[ {Fz_{opt} - CI_{Fz} } \right] < Fz_{exp} < \left[ {Fz_{opt} + CI_{Fz} } \right] \to \left[ {160.867 - 76.83} \right] < 170 < \left[ {160.867 + 76.83} \right] = 84.037 < 170 < 237.697$$

15 $$\left[ {Ra_{opt} - CI_{Ra} } \right] < Ra_{exp} < \left[ {Ra_{opt} + CI_{Ra} } \right] \to \left[ {2.996 - 0.428} \right] < 2.851 < \left[ {2.996 + 0.428} \right] = 2.568 < 3.250 < 3.424$$

16 $$\left[ {DD_{opt} - CI_{DD} } \right] < DD_{exp} < \left[ {DD_{opt} + CI_{DD} } \right] \to \left[ {0.050 - 0.0039} \right] < 0.049 < \left[ {0.050 + 0.0039} \right] = 0.0461 < 0.049 < 0.0539$$

17 $$\left[ {CD_{opt} - CI_{CD} } \right] < DS_{exp} < \left[ {DS_{opt} + CI_{CD} } \right] \to \left[ {0.016 - 0.0028} \right] < 0.017 < \left[ {0.016 + 0.0028} \right] = 0.0132 < 0.017 < 0.0188$$

The fact that the results obtained from the confirmation experiments remain within the calculated CI limits confirms the statistical validity of the optimization performed using the Taguchi method at a 95% confidence level.

## Conclusions

This study demonstrated a comprehensive route to manufacture and drill Direct Hot-Pressed (DHP) Al/SiC composites while systematically optimizing hole quality using the Taguchi method. Al/SiC composites containing 0, 5, and 10 vol.% SiC were successfully consolidated at 600 °C under 35 MPa for 5 min, producing sound compacts with homogeneous reinforcement distribution. SEM observations confirmed that the relatively coarse Al powders and fine, angular SiC particles facilitated efficient packing and dispersion, increasing the matrix–reinforcement interfacial area. Density measurements revealed a clear trade-off: although theoretical density increased with SiC addition, experimental and relative densities decreased (from 98.58% for Al to 96.23% for Al/10SiC), indicating that higher reinforcement contents hinder full densification and promote porosity. Despite this, microhardness increased markedly with SiC fraction (56.7 HV for Al to 75.8 HV for Al/10SiC), showing that particle strengthening dominates over the negative effect of increased porosity. XRD patterns verified the presence of α-Al and SiC peaks and, critically, the absence of detrimental interfacial reaction products such as Al_4_C_3_, confirming chemical stability under the selected DHP conditions. Overall, these outcomes indicate that DHP can yield microstructurally uniform Al/SiC composites with enhanced hardness while preserving interfacial integrity, which is essential for subsequent machining performance.

Experiments were drilled using an L_18_ mixed-level Taguchi orthogonal array to minimize thrust force (Fz), surface roughness (Ra), diameter deviation (DD), and circularity deviation (CD) amounts so that the common issues related to surface integrity and geometry accuracies could be addressed under one platform. The feed rate was found to consistently govern the quality of drilling. According to the S/N analysis and mean response tables, a feed rate of 0.08 mm/rev at medium cutting speeds provided minimum cutting load, limited tool deflection, and ensured stable hole formation. ANOVA proved quantitatively that feed rate was the most common and important factor governing the quality of the hole: feed rate controlled about 53.49% of the Fz variation and 52.58% of the Ra, followed by DD (43.40%) and CD (30.25%). These findings emphasize that, for DHP Al/SiC composites, chip thickness and material removal per revolution govern both mechanical loading and the resulting surface and dimensional quality more strongly than the other investigated factors.

Depending on the target, all stepwise regression control variables behaved differently. Namely, increasing the Vc led to a better surface finish (a noticeable contribution of 13.57% to Ra), a phenomenon perhaps induced by lessened adhesion and more stable chip formation (geometric deviations, particularly DD and CD, weakened, especially because of dynamic impacts, thermal expansion, and the probable effects of runout errors). By bonding, the PA generally enhanced the performance of the drill by imparting stability to the cutting edge and thus reducing smearing, guided by predominant effects on Fz (30.28% contribution) and CD (17.81%). A modulated composite ratio also comprised a key player, introducing an SiC content to contain Ra, DD and CD for improved toll and workpiece dynamics through larger mechanical protection by stiffing the composite way over any plastic flow. Tool and chip observations strongly affirmed said opinions, under which with lower feed, enhancing the reinforcing decreased the built-up edge and increased fragmentation die chip, while higher feed when suitably matched with Al matrix without reinforcing promoted a higher level of adhesion, relatively massive burr, and a long string of continuous ductile chips.

Optimum settings minimizing Fz and Ra were observed as ${Vc}_{2}{f}_{1}{PA}_{3}{RR}_{3}$ (50 m/min, 0.08 mm/rev, 136°, 10 vol.% SiC). However, ${Vc}_{1}{f}_{1}{PA}_{3}{RR}_{3}$ appeared as the optimal condition for DD and CD (25 m/min, 0.08 mm/rev, 136°, 10 vol.% SiC). This clear difference indicates the fact that there is a trade-off when real cutting conditions are taken into account: A higher Vc might improve surface integrity but reduce geometric accuracy. Experimental validation of the Taguchi optimizations performed matched the theoretical results: in the optimal settings for Fz and Ra, the experimental values were 170 N and 3.250 µm (predicted 160.867 N and 2.996 µm consideration), corresponding to 20.76% and 37.43% improvements in S/N respectively. For geometric shape, measurements for DD and CD at parameter settings the same as predicted, Experiment 3, were 0.049 and 0.017 mm, respectively, in good agreement with predicted values. Interval estimation also highlighted the robustness of the optimization. Generally, this research provides an operational and statistical approach that deals with drilling-induced hole quality in high-quality Al-based metal matrix composites containing SiC particles. Results show the feed rate to be the most important factor for surface and geometric properties to take into consideration, whereas both PA and RR give way to some extent. With the achieved values, researchers can make approximations on optimal drilling conditions for the highest possible hole quality in such a manner that Al/SiC composites are included.

Future studies should expand the investigated drilling window by considering broader ranges of cutting conditions (e.g., spindle speed/cutting speed, feed rate, and point angle) and, importantly, the influence of tool material and coating. In particular, using advanced tools such as PCD or TiAlN-coated carbide drills could enable a more wear-resistant cutting interface, thereby providing deeper insight into tool-wear mechanisms and their direct linkage to hole surface integrity and geometric accuracy in abrasive Al/SiC composites. Such studies, supported by quantitative wear measurements, would further strengthen the generalizability of the optimization framework presented in this work.

## Supplementary Information

Below is the link to the electronic supplementary material.

Supplementary Material 1

## Data Availability

Data are available upon request.
